# Is farmland financial innovation narrowing the urban-rural income gap? A cross-regional study of China

**DOI:** 10.1371/journal.pone.0269503

**Published:** 2022-06-03

**Authors:** Ting Li, Jing-Ya Li

**Affiliations:** 1 Department of Economics, Tongling University, Tongling, Anhui, China; 2 Department of Economics, Ocean University of China, Qingdao, Shandong, China; Gebze Teknik Universitesi, TURKEY

## Abstract

Over the past four decades, China’s economy has experienced tremendous economic growth but also a widening urban-rural income gap. Given the dilemma of the urban-rural income gap in China explained by neoclassical equilibrium theory, this paper attempts to provide a new theoretical explanation for the large-income gap between urban and rural areas in China. We select data from 30 provinces(cities) in China over the period from 2006 to 2017 as a sample to investigate whether and how the degree of farmland financial innovation narrows the urban-rural income gap. The results show that the coefficient for farmland financial innovation is significantly negative at the 1% level, signifying that financial innovation can narrow the urban-rural income gap in China. The mediation effect test provides evidence that farmland financial innovation narrows the urban-rural income gap by promoting the permanent migration of the labor force and upgrading the industrial structure. Our results indicate that the government should promote various forms of farmland financial innovation, establish rural property rights transaction system and free farmers from deep farmer-land attachment to realize permanent labor migration.

## 1. Introduction

Based on the equilibrium theory of standard neoclassical economics, the income gap will lead to factor flow until convergence is achieved [1]. Theoretically, the free flow of the labor force will narrow the income gap, and per capita income will converge over time. However, according to the National Bureau of Statistics (NBS) in China, the urban-rural income gap (URIG) has maintained a high-level in recent years. From 2010 to 2018, the URIG fluctuated around 2.7 and in 2019, it still hit 2.64. It seems that the inflection point of Kuznets inverted U-curve has not occurred. The theory of neoclassical economics suggests that when the economic system is out of balance, the system will adjust spontaneously and gradually move toward a new equilibrium. Nevertheless, a series of studies finds that the URIG in China has deviated dramatically since entering this century. Therefore, it seems that the equilibrium theory of neoclassical economics falls into a dilemma of contradiction between theory and reality when explaining the current trend of the URIG in China.

The dual household registration system is undoubtedly the primary exogenous barrier that restricts the free migration of labor, which leads to the differentiation of social security and discriminatory public welfare, resulting in the deterioration of urban and rural residents’ income distribution [2, 3]. Undoubtedly, China’s dual household registration system was an important factor affecting labor migration between urban and rural areas in the early stage of reform and opening-up [4]. However, after entering the 21st century, urban settlements have been completely liberalized in most provinces, and the influence of the hukou system on the flow of labor between urban and rural areas has gradually weakened [5]. However, China’s URIG has not shown a narrowing trend that coincides with the reform of the hukou system [6, 7]. This fact indicates that the hukou system has limited explanatory power for the current large-income gap between urban and rural residents in China, and it is essential to seek a new theoretical foundation to explain the factors restricting labor mobility [8, 9].

Economists have long noticed that rural-urban migration in China shows unique spatial characteristics given China’s special political system [10, 11]. Urbanization has witnessed many farmers migrating to cities, but the main space form of rural labor migration is a nonpermanent migration mode between urban and rural areas [12, 13]. In 2011, an authoritative research by the Development Research Center of the State Council found that only 8.13% of the surveyed migrant workers planned to settle permanently in their working city. There is a super-economic attachment between farmers and land. Farmers cannot permanently settle in the city due to the lack of "threshold" expenses to settle there, so they have to choose nonpermanent migration between urban and rural areas. Long-term migrant workers bear the risk of losing land, and the instability of land use rights may ultimately inhibit labor migration [14]. However, some studies find that the delinking of land property rights and land use rights can stimulate labor migration [15, 16]. By constructing an intertemporal economic model, Valsecchi confirmed the instability of land use rights and non-permanent migration behavior have tight ties [16].

When the relative wages between rural and urban areas change, farmers in private land ownership countries can sell their land and obtain enough funds to settle down in the city [17]. However, at present, China’s rural land is collectively owned, and farmers only have contract rights for a certain period, not indefinitely [18]. As a result, farmers cannot sell, transfer or mortgage their land to use for cost of living in the city, which is very common in private land ownership countries. Due to immovable and unvital land, farmers cannot leave rural areas and settle in cities permanently, so they can only choose nonpermanent migration between urban and rural areas. It is the lack of farmland financial innovation and the resulting farmer-land attachment that explains why labor flow has failed to narrow the income gap between residents of urban and rural areas in China.

Existing studies on rural land finance and urban-rural income can be broadly be summarized into three main viewpoints. One viewpoint emphasizes the poverty reduction effect of farmland financial development. Oded and Joseph, Banerjee and Duflo constructed multisectoral model from the perspective of career choice and human resource, finding that the development of farmland finance will narrow the income gap between urban and rural areas [19, 20]. Jalilian and Kirkpatrick used panel data from 42 developing countries and their empirical results showed that financial development is useful to narrowing the urban-rural income gap [21]. This finding was also verified in Honohan’s research based on data from China, South Korea, Russia, and the United Kingdom [22]. Another viewpoint emphasizes the threshold effect of farmland financial development from the financial gain inequality theory, which means that financial innovation will further widen the gap between urban and rural areas especially in the economically underdeveloped stage. De Haan and Sturm used a panel fixed effects model for a sample of 121 countries covering 1975–2005, and their results suggested that all finance variables increase income inequality [23]. The third viewpoint holds that there is a nonlinear relationship between financial development and the urban-rural income distribution. Greenwood and Jovanovic confirmed that the relationship between financial development and income distribution presented an inverted "U" shape. In other words, in the transition from a primitive economy to a developed one, a nation passes through a stage in which the distribution of wealth among the rich and poor widens [24].

The above studies do not sufficiently consider the particularity of agricultural land finance innovation and lack an in-depth discussion of the mechanism. This paper is thus motivated to address the following questions: Can farmland financial innovation narrow the urban-rural income gap? What are the potential transmission channels through which the farmland financial innovation narrows the urban-rural income gap? The unique land system and economic background make China a particularly interesting and suitable case for analyzing the above questions.

Selecting interprovincial panel data from 2006 to 2017 as a research sample, our study shows that farmland financial innovation has a significant negative impact on the urban-rural income gap. The mediation effect provides evidence that farmland financial innovation can narrow the urban-rural income gap by promoting the permanent migration of the labor force and upgrading the industrial structure. To verify the reliability of our empirical findings, we relaunch a series of auxiliary verifications that provide further support for our main finding.

The marginal contributions of this paper are mainly threefold. First, given the dilemma of the urban-rural income gap in China explained by neoclassical equilibrium theory, this paper provides a new theoretical explanation for the large-income gap between urban and rural areas in China from the perspective of land financial innovation. Second, existing studies mainly focus on the direct effect of farmland financial development, and the important mechanism of farmland financial innovation on urban-rural income gap has not been explored. This paper pays attention to China’s unique land system and discovers potential mechanisms from the perspective of labor migration and industrial upgrading, which further enriches the existing literature. Third, we have innovated the measurement of the "agricultural financial innovation" index from two dimensions, namely, the permeability of farmland financial services and the use effectiveness of farmland financial services. Therefore, our paper also has reference value for future research in the construction of indices.

## 2. Theoretical framework

Farmland finance is an important part of rural finance, and greatly affects urban and rural residents’ income distribution. Regarding the impact of rural financial development on the urban-rural residents’ income inequality, economists put forward the poverty reduction effect and threshold effect from the perspective of financial breadth and depth respectively, which have opposite effects on the urban-rural income gap.

### 2.1 Direct effect

#### 2.1.1 Poverty reduction effect

From the perspective of financial breadth, in theory, rural financial development enables more farmers to enjoy financial services, alleviates the problem of agricultural financing, and increases farmers’ income by improving agricultural output efficiency to narrow the urban-rural income gap, which is called the poverty reduction effect. Through the development of microfinance and the provision of microcredit, savings, exchange and payment, insurance and other transaction services, rural low-income people who are excluded from traditional financial services can be included in the scope of rural financial services. Therefore, they can share the welfare improvement brought about by economic growth, which is useful for narrowing the income gap between residents of urban and rural areas [25].

#### 2.1.2 Threshold effect

From the perspective of financial depth, financial deepening means that financial services are more concentrated in high-end users, providing more comprehensive services for high-income people. This lies in the provision of financial products and services that need to pay a certain fixed cost. Only high-income people can rely on their capital and credit accumulation to afford this part of the cost, enjoy financial services, and invest in projects with higher profit. Financial credit is no longer a simple monotonically increasing function of income but a "threshold" requirement for minimum income. Many theoretical and empirical studies have shown that under the condition of imperfect information in the credit market, initial wealth differences have a systematic impact on future income distribution, and moral hazard and credit constraints are the root causes of capital market failure [26].

In summary, the poverty reduction effect and threshold effect of rural land financial development have opposite effects on the urban-rural income gap, and the final direction depends on the relative strength of the two effects. Based on the above analysis, this paper proposes the first competitive hypothesis as follows:

Hypothesis 1–1: The poverty reduction effect of rural land financial development is greater than the threshold effect, and farmland financial innovation is useful to narrow the urban-rural income gap.

Hypothesis 1–2: The threshold effect of rural land financial development is greater than the poverty reduction effect, and farmland financial innovation will expand the urban-rural income gap.

### 2.2 Indirect effect (mediation effect)

Indirect effects are bridged by mediating variables that link farmland finance and the urban-rural income gap. The research on the mechanism between variables is the focus of theoretical analysis and the important content of the empirical test. The following analysis of mediation effects using labor migration and industrial structure upgrading as mediating variables theoretically clarifies the potential transmission channels through which farmland finance impacts the urban-rural income gap.

(1) Farmland Financial Innovation-Labor Migration-Residents Income Gap between Urban and Rural Areas

Economists have noticed for a long time that, different from the permanent migration model of rural labor in private land ownership countries, the migration of urban and rural labor in China is manifested in one-way flow and nonpermanent migration. How to realize the permanent migration of the labor force and eliminate the attachment between farmers and land has become a crux to narrow the income gap between urban and rural residents. Farmland financial innovation capitalizes the future income of farmers from contracted land, and securitization can activate the financial attributes of farmland assets. Farmers can realize their land rights in the secondary market and obtain valuable funds for settling in the city, which helps overcome the constraint of "threshold" funds. At the same time, the financialization of farmland has transformed farmers’ possession of land from physical form to monetary form, eliminating the attachment between farmers and land and then realizing the recombination of labor and capital in migration cities. After the establishment of the mechanism of permanent labor migration and the two-way flow of urban and rural factors, the most important marginal equalization effect in the process of factor income convergence can be established. Rural low-income people can migrate to cities to increase the marginal income of rural residents, while urban high-income people can also bring funds for rural development to reduce the marginal income of urban residents and gradually equalize the income between urban and rural residents marginally.

In summary, regarding the indirect impact of farmland financial innovation with labor migration as the mediating variable on the urban-rural income gap, the following hypothesis is proposed for testing.

Hypothesis 2: Farmland financial innovation promotes the permanent migration of the rural labor force, and the flow of population reduces the income gap between urban and rural areas.

(2) Farmland Financial Innovation-Industrial Structure Upgrading- Residents Income Gap between Urban and Rural Area

Economic development is not only the increase in total product but also a process of continuous optimization of the industry structure and income structure. Financial development acts on the adjustment and optimization of industrial structure through the endogenous driving forces of demand and supply in the process of economic transformation [27]. Endogenous driving of demand refers to the adjustment of the consumption structure due to the different income elasticities of product demand, which upgrades the industrial structure from the demand side [28]. When the total amount of social capital is fixed, financial development can speed up the flow of capital to high-efficiency economic sectors, promote economic growth and increase per capita income, and then affect the industrial structure through the "income effect". Endogenous driving of supply, also known as the "substitution effect", means that the relative price of products among departments will change due to the difference in the technological progress rate, which will promote the flow of funds from low-efficiency departments to high-efficiency departments [29]. With technological innovation as the intermediary, many theoretical and empirical studies have confirmed that financial development plays a significant role in upgrading industrial structure [30, 31]. The change in industrial structure will inevitably bring about the change in employment structure, which will finally affect the urban-rural income gap. The Kuznets effect shows that in the process of economic development, the difference in comparative labor productivity (that is, the proportion of income in an industry divided by the proportion of employment in the industry) of the three industries continues to converge, which is also the most direct proof that the industrial structure affects the urban-rural income gap [32].

In summary, regarding the indirect impact of farmland financial innovation with industrial structure upgrading as the mediating variable on the urban-rural income gap, the following hypothesis is proposed for testing.

Hypothesis 3: Farmland financial innovation promotes the upgrading of industrial structure, and optimizing the industrial structure is useful in narrowing the urban-rural income gap.

## 3. Materials and methods

### 3.1 Measure of core variables

#### 3.1.1 Measuring farmland financial innovation

In this section, we establish the index system of farmland financial innovation from the two dimensions of service permeability and use effectiveness.

(1) Permeability of Farmland Financial Services

Permeability is the basic layer of rural financial development, which refers to the breadth and density of financial services provided by agricultural financial institutions to the "agriculture, rural areas and farmers" and reflects the degree of rural financial innovation from the supply side. The permeability of farmland financial services can be further decomposed into geographic and demographic permeability, which reflect the breadth and density of financial services, respectively. The dimension of geographic permeability can be proxied by three indices, including the number of agricultural financial institutions per 10,000 square kilometers, the number of employees in agricultural financial institutions per 10,000 square kilometers, and the total agricultural financial assets per 10,000 square kilometers. The dimension of population permeability can be proxied by three indices, including the number of agricultural financial institutions per 10,000 people, the number of employees in agricultural financial institutions per 10,000 people, and the total agricultural financial assets per 10,000 people.

(2) Use Effectiveness of Farmland Financial Services

The effectiveness of farmland financial services refers to the application effect of agricultural financial services to the "agriculture, rural areas and farmers", which reflects the degree of rural financial innovation from the demand side. The effectiveness of financial services can be decomposed into two dimensions: population effectiveness and income effectiveness. The dimension of population effectiveness is proxied by the per capita agricultural loan balance. The dimension of income effectiveness is proxied by the proportion of per capita agricultural loans to per capita income. Table 1 represents the three-level index system of farmland financial innovation in China.

**Table 1:** Evaluation index system of farmland financial innovation in China.

First level index	Second level index	Third level index
Permeability of farmland financial services	Geographic dimension	Number of agricultural financial institutions per 10,000 square kilometers
		Number of employees in agricultural financial institutions per 10,000 square kilometers
		Total agricultural financial assets per 10,000 square kilometers
	Demographic dimension	Number of agricultural financial institutions per 10,000 people
		Number of employees in agricultural financial institutions per 10,000 people
		Total agricultural financial assets per 10,000 people
Use effectiveness of farmland financial services	Population dimension	Per capita agricultural loans balance
	Income dimension	The proportion of per capita agricultural loans to per capita income

#### 3.1.2 Calculation of farmland financial innovation

Based on the evaluation index system of farmland financial innovation in China (see Table 1), the weights for indices were calculated by the coefficient of variation method (CV), which can be used to calculate the FFI. The calculation formula is as follows:

$$V_{i}=\frac{\sigma_{i}}{\bar{X_{i}}}\;i=1,2,\cdots ,n$$ (1)

Here, *σ*_*i*_ and ${\bar{X}}_{i}$ denote the standard deviation and mean value of the relative index above, respectively, and then the weight of each index determined according to the CV method is:

$$w_{i}=\frac{V_{i}}{\sum_{i=1}^{n}V_{i}}\;i=1,2,\cdots ,n$$ (2)

Here, in 0≤*w*_*i*_≤1, the higher the *w*_*i*_ value is, the more important the index is in measuring farmland financial innovation. Table 2 presents the weight of the third-level indices in Table 1.

**Table 2:** The weight of third-level indices of farmland financial innovation.

Index	Weight
Number of agricultural financial institutions per 10,000 square kilometers	0.0880
Number of employees in agricultural financial institutions per 10,000 square kilometers	0.2356
Total agricultural financial assets per 10,000 square kilometers	0.1887
Number of agricultural financial institutions per 10,000 people	0.0420
Number of employees in agricultural financial institutions per 10,000 people	0.1654
Total agricultural financial assets per 10,000 people	0.1164
Per capita agricultural loans balance	0.0906
The proportion of per capita agricultural loans to per capita income	0.0773

Considering that the indices with different dimensions cannot be compared directly, the linear threshold method is used to normalize each index. The specific calculation formula of each adjusted index is as follows:

$$D_{i}=w_{i}\times \frac{X_{i}-m_{i}}{M_{i}-m_{i}}$$ (3)

*X*_*i*_ is the actual value of *i*, the index of a province in a year, *M*_*i*_ is the maximum value of the *i* index, and *m*_*i*_ is the minimum value of the *i* index. The value range of the *D*_*i*_ index is [0,*w*_*i*_]. The degree of farmland financial innovation in a province can be expressed in the form of a dimension vector *V*_*i*_ = [*D*_1_,*D*_2_⋯*D*_*n*_]. In general, farmland financial innovation *FFI* can be synthesized by the Euclidean space distance between the measured value and the ideal value of each province in each year. The calculation formula is as follows:

$$FFI=1-\frac{\sqrt{{\left(w_{1}-D_{1}\right)}^{2}+{\left(w_{2}-D_{2}\right)}^{2}+\cdots {\left(w_{n}-D_{n}\right)}^{2}}}{\sqrt{w_{1}^{2}+w_{2}{}^{2}+\cdots w_{n}^{2}}}$$ (4)

#### 3.1.3 Measuring labor migration

Different from the two-way flow of the labor force between urban and rural areas in developed countries and most developing countries, China’s labor migration is a one-way flow of the rural population to urban areas. This special one-way population migration model provides us with the possibility to calculate the flow of the urban and rural labor force. We can use the difference in the number of rural populations in the adjacent years to estimate the number of labor migrations in that year. To improve the accuracy of the estimation, it is also necessary to consider the impact of natural population changes, such as birth rate and death rate, in the estimation formula. The rural deceased population who did not participate in the migration should be subtracted, while the newborn population who participated in the migration should be added. Fortunately, the existing statistical data can provide complete panel data of the rural population, birth rate and mortality rate of each province since 2005. On this basis, labor migration can be calculated. The specific estimation formula is as follows: (1) The number of labor migrations in a province in a certain year (*Trans*_*t*_) equals the number of rural populations in the province in the previous year (*Population*_*t*+1_) minus the number of rural populations in the province in that year (*Population*_*t*_), minus the number of rural deaths in the province in that year (*Deaths*_*t*_) and adding the rural newborn population in that year (*Newborn*_*t*_). (2) The rural deceased population in a province in a certain year(*Deaths*_*t*_) equals the number of rural populations in the province in that year(*Population*_*t*_) multiply by the rural population death rate of the province in that year(*Deathsrate*_*t*_). (3) The number of rural-born population in a province in a certain year(*Newborn*_*t*_) equals to the number of rural populations in the province in that year (*Population*_*t*_) multiply by the birth rate of the rural population in that year(*Birthrate*_*t*_). The formula for measuring the amount of labor migration is as follows:

$$Trans_{t}=Population_{t+1}-Population_{t}-Deaths_{t}+Newborn_{t}$$ (5)

$$Deaths_{t}=Population_{t}\times Deathsrate_{t}$$ (6)

$$Newborn_{t}=Population_{t}\times Birthrate_{t}$$ (7)

### 3.2 Econometric specifications

To verify the correlation between farmland financial innovation and the urban-rural income gap, we employ the following baseline multivariate econometric specification:

$$URIG_{i,t}=\alpha_{0}+\alpha_{1}FFI_{i,t}+\alpha_{2}ISS_{i,t}+\alpha_{3}ER_{i,t}+\alpha_{4}TR_{i,t}+\alpha_{5}RT_{i,t}+\alpha_{6}Trans_{i,t}+\mu_{i}+\varepsilon_{i,t}$$ (8)

where Eq (4), The subscript *i* represents a province (city) in China, and the period is represented by *t*. To separately control province-specific heterogeneous characteristics, the individual fixed effect *μ*_*i*_ is included, and *ε*_*i*,*t*_ is the residual sum of squares. The dependent variable *URIG* represents the urban-rural income gap, measured by the income ratio between urban and rural areas. The core independent variable *FFI* denotes farmland financial innovation, and its detailed calculation is shown above. The mediation variable *Trans* proxies for labor migration, and the variable *ISS* proxies for industrial structure upgrading. In addition, this paper also includes several control variables, namely, (1) the rural Engel coefficient (*ER*); (2) the per capita rural fixed telephone number (*RT*); and (3) the degree of dependence on foreign trade (*TR*). Table 3 summarizes the definitions and constructions of the variables used in the regressions. The coefficient *α*_1_ illustrates the effect of farmland financial innovation on the urban-rural income gap. The sign *α*_1_ is unclear based on our theoretical analysis.

**Table 3:** Variable definition and constructions.

Variable	Variable definition	Constructions
*FFI*	farmland financial innovation	see above
*ISS*	industrial structure	The ratio of production value of tertiary industry over total GDP
*URIG*	urban-rural income gap	The ratio of per capita disposable income of urban residents over per capita net income of rural residents
*TR*	degree of dependence on foreign trade	The ratio of the total export-import volume over GDP
*ER*	rural Engel coefficient	The ratio of expenditure on necessities for rural residents over total expenditure
*RT*	rural per capita fixed telephone number	Number of telephones per 10000 rural residents
*Trans*	labor migration	see above
*UR*	urbanization rate	The ratio of urban resident population over total population

### 3.3 Data and variables

We select 30 provinces (cities) in China (Tibet Province is not included) as the research sample. All raw data are collected at an annual frequency from 2006 to 2018. The urban-rural income gap index is obtained from China Statistical Yearbook. The sub-indicators of the financial farmland innovation index are manually collected from the China Finance Statistical Yearbook and China Rural Finance Statistical Yearbook. Other provincial control variables are retrieved from the statistical yearbook of provinces and cities and the China Urban-Rural Construction Statistical Yearbook. The GDP data are retrieved from the website http://www.stats.gov.cn/. We have described the data processing in detail in Section 3.1 above. Given that a differential processing for the adjacent year is carried out in measuring the annual labor migration of each province, one year of data will be lost in the time series. Therefore, we finally collect panel data of 30 provinces from 2006 to 2017 in our regression model, with a total of 360 observations.

Table 4 shows the descriptive statistics of each relevant variable above. It can be seen from the table that the minimum value of farmland financial innovation is 0.004, and the maximum value is 0.742, indicating that farmland financial innovation varies greatly between different provinces.

**Table 4:** Summary statistics for main variables.

Variable	Mean	Std.Dev.	Min	P25	Median	P75	Max
*URIG*	2.857	0.544	1.845	2.444	2.766	3.141	4.594
*FFI*	0.081	0.109	0.004	0.032	0.051	0.078	0.742
*ISS*	0.428	0.1	0.256	0.363	0.406	0.468	0.806
*ER*	0.382	0.068	0.247	0.33	0.377	0.429	0.56
*TR*	0.309	0.374	0.017	0.091	0.14	0.357	1.721
*RT*	0.131	0.115	0.008	0.065	0.100	0.151	0.789
*Trans*	4.53	1.37	-1.833	3.763	4.953	5.496	6.505
*GDP*	9.409	0.944	6.475	8.889	9.529	10.046	11.404

Notes: The Table shows the descriptive statistics of the main variables in this research.

## 4. Empirical results and discussions

In this section, we conduct regression analysis based on Eq (8) to investigate the impact of farmland financial innovation on the urban-rural income gap. In addition, we examine the possible mechanisms through which farmland financial innovation affects the urban-rural income gap. Finally, we perform robustness tests by controlling for macroeconomic factors, increasing control variables and applying an alternative metrology method.

### 4.1 Baseline analysis

The Hausman test is employed to determine whether a random or fixed effects regression should be applied in the study. The result shows that the chi-square statistic is 71.46, implying that the fixed effect model should be applied. Table 5 reports the baseline estimation results based on Eq (4). To enhance the credibility of the estimation results, the control variables are gradually added to the baseline regression model. As shown in Column (6) of the table, the coefficient of farmland financial innovation (*FFI*) is -2.105, which is significant at the 1% level after adding all the control variables. As such, Hypotheses 1–1 that we presented earlier can be verified. This result indicates that the poverty reduction effect of farmland financial innovation is greater than the threshold effect; in other words, farmland financial innovation can prominently narrow the urban-rural income gap. This finding is in line with the research of Jalilian and Kirkpatrick, Honohan, Jeanneney and Kpodar [21, 22, 25].

**Table 5:** Results of the baseline regression.

	(1)	(2)	(3)	(4)	(5)	(6)
	*URIG*	*URIG*	*URIG*	*URIG*	*URIG*	*URIG*
*FFI*	-4.131**	-2.596	-0.165	-1.991	-2.139*	-2.105***
	(-2.09)	(-1.25)	(-0.17)	(-1.70)	(-1.96)	(-4.01)
*ISS*		-2.044**	-0.282	-0.434	-0.510	-0.531*
		(-2.46)	(-0.66)	(-1.00)	(-1.19)	(-1.72)
*ER*			4.149***	3.826***	3.928***	3.941***
			(9.42)	(8.88)	(8.80)	(14.71)
*TR*				-0.712**	-0.722**	-0.721***
				(-2.52)	(-2.55)	(-5.88)
*RT*					-0.417	-0.419
					(-0.98)	(-1.42)
*Trans*						-0.005
						(-0.26)
*_cons*	3.192***	3.944***	1.407***	1.964***	2.027***	2.053***
	(19.91)	(16.62)	(5.89)	(6.60)	(6.79)	(8.81)
*IE*	YES	YES	YES	YES	YES	YES
*Adj*-R^2^	0.219	0.282	0.598	0.635	0.638	0.638
*N*	360	360	360	360	360	360

Notes:

*, ** and *** indicate significance at 10%, 5%, and 1% confidence level respectively. The numbers in the parenthesis are corresponding t-values. IE means individual effect.

In addition, we also find that industrial structure upgrading (*ISS*) can significantly reduce the urban-rural income gap (*URIR*). Although labor migration (*Trans*) has a negative impact on the urban-rural resident income gap (*URIR*), the coefficient is only -0.005, and its t-statistic is very small, failing to pass the significance level test. The potential transmission channels through which farmland financial innovation impacts the urban-rural income gap still require further examination.

### 4.2. Exploring the underlying mechanisms

#### 4.2.1. Labor migration as the channel

The baseline regression model confirmed that farmland financial innovation has a negative effect on the urban-rural income gap. However, how does farmland financial innovation affect the urban-rural residents’ income ratio; in other words, what are the potential transmission channels in this relationship? Based on the previous theoretical analyses in the indirect effect hypothesis, farmland financial innovation can narrow the urban-rural income gap by eliminating farmer-land attachment, promoting the permanent migration of the labor force and upgrading the industrial structure. We, therefore, have two potential mediating variables, namely, labor migration and industrial structure upgrading. Referring to the mediation effect test procedure of Baron and Kenny, we examine two potential transmission channels in this section and construct the equations as follows [33].


$$Trans_{i,t}/ISS_{i,t}=\beta_{0}+\beta_{1}FFI_{i,t}+\beta Controls_{i,t}+u_{i}+\varepsilon_{i,t}$$ (9)

$$URIR_{i,t}=\gamma_{0}+\gamma_{1}FFI_{i,t}+\gamma_{2}Trans_{i,t}/ISS_{i,t}+\gamma Controls_{i,t}+u_{i}+\varepsilon_{i,t}$$ (10)

The corresponding results are reported in Table 6. Column (1) investigates the effect of *FFI* on the mediating variable *Trans*. The coefficient *FFI* is 6.271, which is significantly positive at the 5% level and indicates that farmland financial innovation will accelerate the flow of the labor force between urban and rural areas. In Column (2), the independent variable *Fin* and the mediating variable *Trans* are included to test the joint effects on the urban-rural income gap (*URIR*). The coefficient of *FFI* is significantly negative at the 10% level, and the coefficient of *Trans* is negative, consistent with our theoretical expectation. However, Column (2) further shows that the coefficient fails to pass the significance test. Therefore, the mediation impact should be further validated using the Sobel test. The results of the Soble test show that the Z-statistic value is -2, which is significant at the level of 5%. The partial mediating effect is verified, and the mediation effect accounts for nearly 40%. Thus, this two-step analysis supports the interpretation that labor migration works as the mechanism behind farmland financial innovation and the urban-rural income gap in China; that is, Hypotheses 2 is verified. This result is consistent with the research of Xie, who hold that incomplete land transfer rights restrict the asset attributes and liquidity of land, makes it difficult for migrants to settle permanently in the city [9].

**Table 6:** Results of the transmission channels.

	(1)	(2)	(3)	(4)
	*Trans*	*URIG*	*ISS*	*URIG*
*FFI*	6.271**	-2.105*	0.389***	-2.105***
	(2.10)	(-1.98)	(4.23)	(-4.01)
*ISS*	-3.824	-0.531		-0.531*
	(-1.67)	(-1.25)		(-1.72)
*ER*	2.344*	3.941***	-0.207***	3.941***
	(1.90)	(8.92)	(-4.42)	(14.71)
*TR*	0.175	-0.721**	-0.035	-0.721***
	(0.49)	(-2.55)	(-1.57)	(-5.88)
*RT*	-0.361	-0.419	-0.172***	-0.419
	(-0.14)	(-0.97)	(-3.31)	(-1.42)
*Trans*		-0.005	-0.017***	-0.005
		(-0.16)	(-4.79)	(-0.26)
_cons	4.758***	2.053***	0.587***	2.053***
	(4.83)	(5.62)	(22.23)	(8.81)
*IE*	YES	YES	YES	YES
*Adj*-R^2^	0.145	0.638	0.473	0.638
*N*	360	360	360	360

Notes:

*, ** and *** indicate significance at 10%, 5%, and 1% confidence level respectively. The numbers in the parenthesis are corresponding t-values. IE means individual effect.

#### 4.2.2. Industrial structure upgrading as the channel

To test whether industrial structure upgrading works as another channel through which farmland financial innovation affects urban-rural residents’ income ratio, we first provide empirical evidence to show that *FFI* does promote industrial upgrading. The corresponding results are shown in Column (3) of Table 6. More specifically, the coefficient *FFI* is 0.389, which is significantly positive at the 1% level. Then we perform the two-step test on Eq (10), investigating the impact of the independent variable (*FFI*) on mediating variable (*ISS*). Both the independent variable (*FFI*) and the mediating variable (*ISS*) are included in the estimation. From Column (4) of Table 6, the coefficient *FFI* is significantly positive at the level of 1%, and the coefficient *ISS* is significantly negative at the level of 10%, indicating that industrial structure upgrading induces the income gap of urban-rural residents in China. Here, we also carry out the Sobel test as above and the result shows that the Z-score is 3.908, which is significant at the level of 1%, signifying that the partial mediation effect is tenable. Thus, this two-step analysis supports the interpretation that industrial structure upgrading works as the mechanism behind farmland financial innovation and the urban-rural income gap in China; that is, Hypotheses 3 is tenable. Financial innovation in agriculture has accelerated industrial upgrading, improving the efficiency of labor markets and factor allocation, thus expanding the ways for rural residents to obtain jobs and increasing their income substantially, which supports the research of Acemoglu and Guerrieri, Rin and Hellmann [30, 31].

### 4.3. Robustness test

It is necessary to test the robustness of the empirical results of the model by controlling macroeconomic factors, increasing control variables and changing parameter estimation methods.

To verify the reliability of our empirical findings, we conduct a battery of sensitivity tests. We relaunch a series of auxiliary verifications by controlling macroeconomic factors, increasing control variables and applying an alternative metrology method, which provide further support for our main finding. We now explain them in the following subsections.

#### 4.3.1. Controlling for macroeconomic factors

Given that controlling for the time fixed effect may lead to multicollinearity problems, referring to Pastor and Veronesi, we add macroeconomic variables including economic development (GDP growth rate) and monetary policy (M2) year-on-year growth rate into the baseline model Eq (1) and then observe whether the impact of farmland financial innovation on the urban-rural income gap changes significantly after controlling for macroeconomic trends [34]. Columns (1) to 2 of Table 7 report the estimation results after adding GDP growth rate and M2 growth rate, respectively. The coefficient *FFI* is still negative and significant at the 1% level, and the coefficients of the control variables also do not change significantly, which further verifies our previous conclusions.

**Table 7:** Robustness test: Controlling macroeconomic factors.

*variables*	(1)	(2)
	*URIG*	*URIG*
*FFI*	-1.411***	-1.423***
	(-2.72)	(-2.62)
*ISS*	-0.397	-0.357
	(-1.34)	(-1.17)
*ER*	3.084***	3.593***
	(10.27)	(12.98)
*TR*	-0.768***	-0.584***
	(-6.52)	(-4.67)
*RT*	-0.506*	-0.480*
	(-1.79)	(-1.66)
*Trans*	-0.015	-0.012
	(-0.74)	(-0.59)
*GDP*	3.576***	
	(5.49)	
*M2*		1.056***
		(3.92)
*_cons*	2.009***	1.888***
	(8.99)	(8.14)
*IE*	YES	YES
*Adj*-R^2^	0.669	0.654
N	360	360

Notes:

*, ** and *** indicate significance at 10%, 5%, and 1% confidence level respectively. The numbers in the parenthesis are corresponding t-values. IE means individual effect.

#### 4.3.2. Addressing the potential endogeneity issue

The above analysis of the paper may not have full control over the urban and rural development characteristics of each province, which may lead to missing variables. To eliminate the potential impact of endogeneity problems on the panel regression model, we continue to add rural per capita education years (*Edu*) and urbanization rate (*UR*) as control variables given the importance of human capital and economic development level in the empirical study of urban and rural residents’ income distribution. Table 8 reports the regression results when gradually adding control variables.

**Table 8:** Robustness test: Increasing possible missing variables.

*variables*	(1)	(2)	(3)	(4)	(5)	(6)	(7)	(8)
	*URIG*	*URIG*	*URIG*	*URIG*	*URIG*	*URIG*	*URIG*	*URIG*
*FFI*	-4.131**	-2.596	-0.165	-1.991	-2.139*	-2.105***	-0.988*	-1.159**
	(-2.09)	(-1.25)	(-0.17)	(-1.70)	(-1.96)	(-4.01)	(-1.82)	(-2.28)
*ISS*		-2.044**	-0.282	-0.434	-0.510	-0.531*	-0.454	-0.219
		(-2.46)	(-0.66)	(-1.00)	(-1.19)	(-1.72)	(-1.54)	(-0.79)
*ER*			4.149***	3.826***	3.928***	3.941***	3.075***	1.434***
			(9.42)	(8.88)	(8.80)	(14.71)	(10.23)	(3.88)
*TR*				-0.712**	-0.722**	-0.721***	-0.673***	-0.525***
				(-2.52)	(-2.55)	(-5.88)	(-5.72)	(-4.68)
*RT*					-0.417	-0.419	-0.523*	-0.578**
					(-0.98)	(-1.42)	(-1.85)	(-2.19)
*Trans*						-0.005	-0.012	0.014
						(-0.26)	(-0.61)	(0.76)
*Edu*							-0.200***	-0.041
							(-5.52)	(-0.99)
*UR*								-3.406***
								(-6.86)
*_cons*	3.192***	3.944***	1.407***	1.964***	2.027***	2.053***	4.046***	4.850***
	(19.91)	(16.62)	(5.89)	(6.60)	(6.79)	(8.81)	(9.54)	(11.72)
*IE*	YES	YES	YES	YES	YES	YES	YES	YES
*Adj*-R^2^	0.219	0.282	0.598	0.635	0.638	0.638	0.669	0.711
N	360	360	360	360	360	360	360	360

Notes:

*, ** and *** indicate significance at 10%, 5%, and 1% confidence level respectively. The numbers in the parenthesis are corresponding t-values. IE means individual effect.

Column (8) of Table 8 presents the result of the regressions including *Edu*, *UR* and other relevant control variables. Compared with Column (6), we find that although the coefficient (absolute value) of *FFI* shows a slight decrease, the sign and significance of *Fin* show no obvious change. As such, Hypotheses 1–1 that we presented earlier can be verified again. Column (7) of Table 8 further shows that the coefficient *Edu* is significantly negative at the 1% level, implying that improving farmers’ education level is an essential part of narrowing the income gap between urban and rural areas. Column (8) of Table 8 further shows that the coefficient *UR* is also significantly negative at the 1% level. This confirms that urbanization is part of an inclusive and healthy economic growth strategy, which is in line with the theoretical expectation.

#### 4.3.3. Alternative metrology method

In this section, to address potential heteroscedasticity and autocorrelation problems, *MLE* estimation methods are used for the robustness tests. Maximum likelihood estimation (*MLE*) is not sensitive to the standardization of parameters and models. Although the *MLE* estimator is asymptotically distributed, it is still more effective and has less estimation bias than moment estimation in relatively small samples.

The corresponding regression results are shown in Columns (1) to (6) of Table 9. With this model specification, a 1% increase in *FFI* is significantly associated with a 1.171% decrease in *URIG* after all control variables are included, which further strengthens our previous findings.

**Table 9:** Robustness test: Alternative metrology method.

	(1)	(2)	(3)	(4)	(5)	(6)
	*URIG*	*URIG*	*URIG*	*URIG*	*URIG*	*URIG*
*FFI*	-3.603***	-2.222***	-0.332	-1.207***	-1.211***	-1.171***
	(-8.95)	(-4.71)	(-0.89)	(-2.99)	(-2.99)	(-2.87)
*ISS*		-1.944***	-0.268	-0.268	-0.280	-0.332
		(-5.22)	(-0.90)	(-0.94)	(-0.97)	(-1.11)
*ER*			4.082***	4.062***	4.086***	4.108***
			(16.47)	(16.95)	(16.04)	(16.00)
*TR*				-0.523***	-0.519***	-0.526***
				(-5.50)	(-5.37)	(-5.43)
*RT*					-0.075	-0.086
					(-0.28)	(-0.32)
*Trans*						-0.013
						(-0.65)
*_cons*	3.149***	3.870***	1.441***	1.681***	1.686***	1.757***
	(32.99)	(22.90)	(7.28)	(8.42)	(8.39)	(7.69)
*sigma_u*	0.486***	0.502***	0.425***	0.414***	0.417***	0.416***
	(7.25)	(7.32)	(7.59)	(7.17)	(7.03)	(7.07)
*sigma_e*	0.258***	0.247***	0.185***	0.177***	0.177***	0.177***
	(25.59)	(25.60)	(25.68)	(25.54)	(25.49)	(25.50)
*N*	360	360	360	360	360	360

Notes:

*, ** and *** indicate significance at 10%, 5%, and 1% confidence level respectively. The numbers in the parenthesis are corresponding t-values.

## 5. Conclusions and policy implications

Farmland financial innovation in China is a process of issuing bonds with the future income of land contract-management rights as a guarantee, thus transforming land into financial products that can be circulated in the financial market without losing land contract rights. Farmland financialization turns immovable land into an asset that can be "carried", liberating farmers from land attachment, and promoting farmers migrating to cities. Hence, we hold that farmland financial innovation is key to narrowing the income gap of residents between urban and rural areas.

This study examines the interaction relationships between farmland financial innovation and the urban-rural income gap using a panel data model over the period of 2006–2017. The main findings are as follows. The results show that the coefficient for farmland financial innovation is significantly negative at the 1% level, signifying that farmland financial innovation can significantly narrow the income gap between urban and rural areas, which also means that the poverty reduction effect of rural finance development is greater than the threshold effect. The findings of the mediating effect indicate that promoting the permanent migration of the labor force and upgrading the industrial structure are the potential transmission channels through which the farmland financial innovation narrows the urban-rural income gap.

Our research has far-reaching policy implications. First, China should promote various forms of farmland financial innovation, such as a mortgage loan of contracted land management rights, shares of land use rights, farmland trusts, which can not only alleviate the contradiction of capital supply and demand in the development of "agriculture, rural areas and farmers", but also help to narrow the current large-income gap between urban and rural areas.

Second, policy-makers must remove the administrative barriers for urban funds to the countryside to change the one-way flow of labor to the city. They should also strengthen the reform of the hukou, education, and social security systems, to promote the permanent migration of middle and low-income rural residents to cities.

Third, it is necessary to establish a rural property rights transaction system, and select economically developed areas to try out a paid withdrawal system for farmland contract management rights if conditions permit. Land contract rights should be separate from the hukou system, so rural residents can migrate to the city and settle without fear of losing their land.

We, of course, acknowledge the deficiencies of our study. Our paper only demonstrates the relationship between financial farmland innovation and the urban-rural income gap from empirical data and we still lack a theoretical model. Future studies may try to develop a dual economy transformation model to understand the mechanism and impact of financial farmland innovation on labor migration and urban-rural inequality. Attention should also be paid to how different financial instruments contribute to reducing income inequality and which financial instrument works best.

## Supporting information

S1 File (ZIP)
