# Supplementary material for: Is farmland financial innovation narrowing the urban-rural income gap? A cross-regional study of China
Source: PLoS One. 2022 Jun 3;17(6):e0269503. doi: 10.1371/journal.pone.0269503 (PMC9165774; doi:10.1371/journal.pone.0269503)
Supplement: Attachment — Submitted filename: Common grammatical errors Checked manuscuipt for plos one.docx [file pone.0269503.s002.docx]

**Is farmland financial innovation narrowing the urban-rural income gap? A cross-regional study of China**

**Ting Li ^1^, and Jinya Li ^2,*^**

^1^ Department of Economics, Tongling University, Tongling 244000,China;liting720427@163.com

^2^ Department of Economics, Ocean University of China, Qingdao 266100, China;

[17865326845@163.com](mailto:17865326845@163.com)

^*^ Correspondence: [17865326845@163.com;](mailto:17865326845@163.com;) Tel: +86-1786-532-6845

# Abstract

Given the dilemma of the urban-rural income gap in China explained by the neoclassical equilibrium theory, we attempt to provide a new theoretical explanation for the high-income gap between urban and rural areas in China. This paper selects data from 30 provinces(cities) in China over the period from 2003 to 2017 as a sample to investigate whether and how the degree of farmland financial innovation narrows the urban-rural income gap. The results show that farmland financial innovation can significantly reduce the urban-rural income gap. Through the heterogeneity analysis, we find that the negative correlation between farmland financial innovation and the urban-rural income gap is more significant in the economic downturn period. Furthermore, the mediation effect provides evidence that farmland financial innovation can narrow the urban-rural income gap through eliminating farmer-land attachment, promoting the permanent migration of the labor force, and upgrading the industrial structure. The results indicate that freeing farmers from the deep farmer-land attachment to realize permanent migration has become an essential measure to narrow the urban-rural income gap in China.

**Keywords:** Farmland financial innovation; Urban-rural income gap; Non-permanent migration of labor force; Farmer-land attachment

# 1 Introduction

Based on the equilibrium theory of standard neoclassical economics, the income gap will lead to factor flowing until achieving convergence (Lucas, 2004). Theoretically, the free flow of the labor force will narrow the factor income gap, and per capita income will converge over time. However, according to the NBS in China, the urban-rural income gap (URIG) has still maintained a high level in recent years. Specifically, from 2010 to 2018, URIG fluctuated around 2.7 and in 2019 it still hit 2.64. It seems that the inflection point of Kuznets inverted U-curve has not arrived. The equilibrium theory of neoclassical economics falls into a dilemma of contradiction between theory and reality when explaining the current trend of URIG in China.

Early literature on explaining high URIG in China holds the following representative viewpoints. The first is the theory of binary economic structure, which advances that binary economic structure is a common economic phenomenon in developing countries and should be the primary reason in explaining high URIG in China (Bose, 1996; Bhaduri and Skarstein, 2004). The second is the theory of urban-biased economic policy. They hold that the unbalanced development strategies including fiscal and tax policy, public goods and transfer payment policy, financial credit policy, and government policy power are the root of deteriorating urban-rural residents income distribution (Lin and Liu, 2003; Fleisher and Chen, 1997; Lu and Chen, 2004). The third is the theory of human capital, which holds that the gap of factor incomes derives from its marginal contribution in production and that human capital investment is the fundamental reason for the difference in factor marginal productivity (Nelson and Phelps,1966; Guo, 2005). However, the binary economic structure seems to be the result of unbalanced urban-rural development instead of the cause of the urban-rural income gap. Urban-biased economic policies can provide theoretical explanations for the reasons for the urban-rural income gap from a certain aspect, but the crux and mutual relationships between the two cannot be clarified. Human capital theory partially grasps the essence of the problem, but with the current nine-year compulsory education has been fully completed and university education has achieved full coverage. This theory seems not to fit the current phenomenon in China very well.

Undoubtedly China’s hukou system (household registration system) was an important factor affecting labor migration between urban and rural areas in the early stage of reform and opening up (Zhou and Cheung, 2017). However, after entering the 21st century, urban settlements have been completely liberalized in most provinces, and the influence of the hukou system on the flow of labor between urban and rural areas has gradually weakened (Shi et al., 2002; Sicular and Yue, 2007). But China's URIG has not shown a narrowing trend that coincides with the reform of the hukou system (Wang and Zhang, 2021; Su et al., 2015). The fact indicates that the hukou system has limited explanatory power for the current high-income gap between urban and rural residents in China, and it is essential to seek a new theoretical foundation to explain the factors in restricting labor mobility (Wan and Li, 2013; Xie, 2014).

Economists have long noticed that rural-urban migration in China shows unique spatial characteristics given China's special political system (Wang and Benjamin, 2019; Ge et al., 2020). Firstly, due to the identity attribute of land contract rights and the government's restrictions on urban capital flowing to the countryside, China's urban and rural labor force is more manifested as a one-way flow from rural to urban areas (Li and Zahniser, 2002). Secondly, urbanization has also experienced many farmers migrating to cities, but the main space form of rural labor migration is a "migratory bird-like" non-permanent migration mode between urban and rural areas (Xie, 2014; Li and Zahniser, 2002; Carrillo, 2004). In 2011, authoritative research by the Development Research Center of the State Council found that only 8.13% of the surveyed migrant workers planned to settle permanently in their working city. There is a super-economic attachment between farmer and land. Although the extension of the land contract period is beneficial to the stability of agricultural production and management, it also strengthens farmer-land attachment objectively. Farmers cannot permanently settle in the city due to the lack of "threshold" expenses to settle in the city， so they have to choose non-permanent migration between urban and rural areas (Xie, 2014; Li and Zahniser, 2002). It is the strong farmer-land attachment and the non-permanent migration of rural labor, that explains why labor flow has failed to narrow the resident’s income gap between urban and rural areas in China.

New economic history studies hold that effective farmland property rights arrangements can increase labor productivity, promote permanent labor migration, and realize dual economic transformation (Valsecchi, 2014; Henderson and Wang, 2007; Wang and Shao, 2019). The formation of strong farmer-land attachment in China's rural areas is not only caused by the accumulation of traditional agriculture civilization for thousands of years but also related to lagged system innovation of current land institutions (Carrillo, 2004; Mullan et al., 2011). Farmland financial innovation is a process of issuing bonds with the future income of land contract-management rights as a guarantee, and transforming it into financial products that can be circulated in the financial market without losing land contract rights (Alain et al., 2015). Farmland financial innovation completely activates the "sleeping" farmland assets, which can greatly alleviate the financing problems of the new agricultural business entities. With the help of the equity capitalization function of financial innovation, it is truly realized that the farmers' possession of land has changed from physical form to value form (North, 1981; Pu, 2014). On the one hand, the farmland financialization turns land into an asset that can be "carried", moving spatially immovable land with the aid of asset securitization, liberating farmers from land attachment, and promoting the integration of migrant population into urban society. On the other hand, the establishment of a compensation mechanism of withdrawing land contract rights can completely cut off the "umbilical cord" between farmers and land, and the crux of farmer-land attachment can be solved fundamentally. In summary, farmland financial innovation is key to narrowing residents’ income gap between urban and rural areas.

This paper is thus motivated to address the following questions based on the context of China: Can farmland financial innovation narrow the urban-rural income gap? What are the potential transmission channels that the farmland financial innovation narrows the urban-rural income gap? Is there any heterogeneity in the impact of farmland financial innovation across different economic cycles? The unique land system and economic background make China a particularly interesting and suitable case for analyzing the above questions.

Selecting inter-provincial panel data from 2006 to 2017 as a research sample, our study shows that farmland financial innovation has a significant negative impact on the urban-rural income gap, which is more intense during the economic recession. The mediation effect provides evidence that farmland financial innovation can narrow the urban-rural income gap through eliminating farmer-land attachment, promoting the permanent migration of the labor force, and upgrading the industrial structure. To verify the reliability of our empirical findings, we re-launch a series of auxiliary verifications from multiple dimensions including replacing of regression method and controlling more potential omitted variables, which provide further support for our main finding. In additional tests, we find significant differential effects of farmland financial innovation on the urban-rural income gap across the economic cycle in two phases. Particularly, in the economic downturn, the role of farmland financial innovation in narrowing the income gap between urban and rural areas is more prominent. So, we propose that enlivening the important asset of farmers' contracted land, freeing farmers from the fixed farmer-land attachment relationship to realize permanent migration, and promoting the equalization of urban-rural residents’ income have become essential measures to narrow the urban-rural income gap in China.

1. **Theoretical Analysis and Research Hypothesis**
   1.1. *Direct Effects*

As an important part of rural finance, land finance has a common impact on urban and rural residents’ income distribution as rural finance. Regarding the impact of rural financial development on urban-rural residents' income inequality, economists put forward the poverty reduction effect and threshold effect respectively from the perspective of financial breadth and depth, which have opposite effects on the urban-rural income gap.

1.1.1. *Poverty Reduction Effect*

From the perspective of financial breadth, in theory, rural financial development enables more farmers to enjoy financial services, alleviate the problem of agricultural financing, and increase farmers' income by improving agricultural output efficiency to narrow resident’s income gap between urban and rural areas, which is called poverty reduction effect. Through the development of micro-finance and the provision of micro-credit, savings, exchange, and payment, insurance, and other transaction services, the rural low-income people who are excluded from the traditional financial services can be included in the scope of rural financial services, so that they can share the welfare improvement brought about by economic growth which is useful for narrowing resident’s income gap between urban and rural areas (Jeanneney and Kpodar, 2011). Jalilian et al. (2002) used panel data from 42 developing countries and Honoban (2001) used data from China, South Korea, Russia, and the United Kingdom to analyze the relationship between financial development, economic growth, and the gap between the rich and the poor, the result has shown that financial development is useful to narrow the income gap, especially in the urban-rural income gap, which is consistent with our hypothesis.

1.1.2. *Threshold Effect*

From the perspective of financial depth, financial deepening means that financial services are more concentrated in high-end users, providing more comprehensive services for high-income people. This lies in that the provision of financial products and services needs to pay a certain fixed cost. Only high-income people can rely on their capital and credit accumulation to afford this part of the cost, enjoy financial services, and invest in projects with higher profits. Financial credit is no longer a simple monotonically increasing function of income, but a "threshold" requirement for minimum income. Greenwood et al. proved that due to the existence of two threshold wealth levels, the relationship between financial development and income distribution presents an inverted "U" shape (Greenwood and Jovanovic, 1990). Subsequently, many theoretical and empirical studies have shown that under the condition of imperfect information in the credit market, initial wealth differences have a systematic impact on future income distribution, and moral hazard and credit constraints are the root causes of capital market failure and persistent income gap (Galor and Zeira, 1993; Aghion and Bolton, 1992).

To sum up, the poverty reduction effect and threshold effect of rural land financial development have opposite effects on the urban-rural income gap, and the final direction depends on the relative strength of the two effects. Based on the above analysis, this paper proposes the first competitive hypothesis as follows:

Hypothesis 1-1: The poverty reduction effect of rural land financial development is greater than the threshold effect, and farmland financial innovation is useful to narrowing the urban-rural income gap.

Hypothesis 1-2: The threshold effect of rural land financial development is greater than the poverty reduction effect, and farmland financial innovation will expand the urban-rural income gap.

1.2. *Indirect Effects (Mediation Effect)*

Indirect effects are bridged by mediate variables, which link the farmland finance and urban-rural income gap. The research on the mechanism between variables is the focus of theoretical analysis and the important content of the empirical test. The following analysis of mediation effects using labor migration and industrial structure upgrading as mediating variables theoretically clarifies the potential transmission channels through which farmland finance impacts the urban-rural income gap.

*2.2.1 Farmland Financial Innovation-Labor Migration-Residents Income Gap between Urban and Rural Area*

Economists have noticed for a long time that, different from the permanent migration model of rural labor in private land ownership countries, the migration of urban and rural labor in china is manifested in one-way flow and non-permanent migration (Li and Zahniser, 2002; Carrillo, 2004; Xie, 2014). It is this semi-urbanization process that has severely weakened the urban-rural factors equalization mechanism and hindered the convergence of urban and rural residents' income. How to realize the permanent migration of the labor force and eliminate the attachment between farmers and land has become a crux to narrow the income gap between urban and rural residents. Farmland financial innovation capitalizes the future income of farmers from contracted land, and it has the dual functions of finance and resource allocation. Securitization can activate the financial attributes of farmland assets. Farmers can realize their land rights in the secondary market and obtain valuable funds for settling in the city, which helps overcome the constraint of "threshold" funds in the process of permanent labor migration. At the same time, the financialization of farmland has transformed farmers' possession of land from physical form to monetary form, cutting off the "umbilical cord" of the connection between farmers and land, eliminating the attachment between farmer and land, and realizing the recombination of labor and capital in migration cities. The farmland finance innovation also provides new investment channels for social investors, provide an opportunity for urban redundant funds to enter the "agriculture, rural areas and farmers" fields, and realizes the optimized allocation of labor, capital, land, and other factors in a larger range of urban and rural space. After the establishment of the mechanism of permanent labor migration and the two-way flow of urban and rural factors, the most important marginal equalization effect in the process of factor income convergence can be established. Rural low-income people can migrate to cities to increase the marginal income of rural residents, while urban high-income people can also bring funds for rural development to reduce the marginal income of urban residents, and gradually fill the income gap between urban and rural residents marginally.

In summary, the indirect impact of farmland financial innovation with labor migration as the mediate variable on the urban-rural income gap, the following mechanism hypothesis is proposed for testing.

Hypothesis 2: Farmland financial innovation promotes the permanent migration of the rural labor force, and the flow of population reduces the income gap between urban and rural areas.

*2.2.2 Farmland Financial Innovation-Industrial Structure Upgrading- Residents Income Gap between Urban and Rural Area*

Economic development is not only the increase of total product but also a process of continuous optimization of the industry structure and income structure. Industrial structure upgrading is an important booster of economic development by optimizing the allocation of scarce production factors, and financial innovation plays an important role. Financial development acts on the adjustment and optimization of industrial structure through the endogenous driving forces of demand and supply in the process of economic transformation (Beck et al., 2000). Demand endogenous driving refers to the adjustment of consumption structure due to different income elasticity of product demand, which upgrades industrial structure from the demand side (Fisher, 1952). When the total amount of social capital is fixed, financial development can speed up the flow of capital to high-efficiency economic sectors, promote economic growth and increase per-capita income, and then affect the industrial structure through the "income effect". Supply endogenous driving, also known as the "substitution effect", refers to that the relative price of products among departments will change due to the difference of technological progress rate, which will promote the flow of funds from low-efficiency departments to high-efficiency departments, and promote the upgrading of industrial structure from the supply side (Baumol,1967). The degree of financial development differs in external financing costs, information-processing mechanisms, and influence on technological innovation. With technological innovation as the intermediary, a large number of theoretical and empirical studies have confirmed that financial development plays a significant role in upgrading industrial structure (Acemoglu and Guerrieri, 2008; Rin and Hellmann, 2002). The change of industrial structure will inevitably bring about the change of employment structure, which will finally affect the urban-rural income gap. Through the statistical analysis of the changes in the distribution structure of national income and labor force in developing countries, Kuznets (1955) found that the proportion of income and labor employment in the agricultural sector are both declining, the proportion of income in the industrial sector first rises and then decreases, the proportion of labor employment remains unchanged, and the proportion of income and employment in the service industry increases asynchronously. Kuznets effect shows that in the process of economic development, the difference of comparative labor productivity (that is the proportion of income in an industry divided by the proportion of employment in the industry) of the three industries continues to converge, which is also the most direct proof that the industrial structure affects urban-rural income gap.

In summary, the indirect impact of farmland financial innovation with industrial structure upgrading as the mediate variable on urban-rural income gap, the following mechanism hypothesis is proposed for testing.

Hypothesis 3: Farmland financial innovation promotes the upgrading of industrial structure, and optimizing the industrial structure is useful in narrowing the urban-rural income gap.

2. **Measuring farmland financial innovation and labor migration**

*3.1 Measuring farmland financial innovation*

In this section, we establish the index system of farmland financial innovation from two dimensions of service permeability and use effectiveness.

(1) Permeability of Farmland Financial Services

Permeability is the basic layer of rural financial inclusion, which refers to the breadth and density of financial services provided by agricultural financial institutions to the "agriculture, rural areas and farmers", and reflects the degree of rural financial innovation from the supply side. The permeability of farmland financial services can be further decomposed into geographic and demographic permeability, which respectively reflects the breadth and density of financial services. The dimension of geographic permeability can be proxied by three indexes including the number of agricultural financial institutions per 10,000 square kilometers, the number of employees in agricultural financial institutions per 10,000 square kilometers, and the total agricultural financial assets per 10,000 square kilometers. The dimension of population permeability can be proxied by three indexes including the number of agricultural financial institutions per 10,000 people, the number of employees in agricultural financial institutions per 10,000 people, and the total agricultural financial assets per 10,000 people.

(2) Use Effectiveness of Farmland Financial Services

The effectiveness of farmland financial services refers to the application effect of agricultural financial services to the "agriculture, rural areas and farmers", which reflects the degree of rural financial innovation from the demand side. The effectiveness of financial services can be decomposed into two dimensions included population effectiveness and income effectiveness. The dimension of population effectiveness is proxied by per capita agricultural loans balance. The dimension of income effectiveness is proxied by the proportion of per capita agricultural loans to per capita income. Table 1 represents the three-level indexes system of farmland financial innovation in China.

Table1. Evaluation indexes system of farmland financial innovation in China.

| First level index | Second level index | Third level index |
| --- | --- | --- |
| Permeability of farmland financial services | Geographic  dimension | Number of agricultural financial institutions per 10,000 square kilometers |
|  |  | Number of employees in agricultural financial institutions per 10,000 square kilometers |
|  |  | Total agricultural financial assets per 10,000 square kilometers |
|  | Demographic  dimension | Number of agricultural financial institutions per 10,000 people |
|  |  | Number of employees in agricultural financial institutions per 10,000 people |
|  |  | Total agricultural financial assets per 10,000 people |
| Use effectiveness of farmland financial services | Population  dimension | Per capita agricultural loans balance |
|  | Income  dimension | The proportion of per capita agricultural loans to per capita income |

To eliminate the influence of dimension, the weights for indexes were calculated by the Coefficient of Variation method (CV). The calculation formula is as follows:

$V_{i}=\frac{\sigma_{i}}{\overline{X_{i}}}$ $i=1,2,\cdots,n$ (1)

Here, $\sigma_{i}$ and $\overline{X}_{i}$respectively denotes the standard deviation and mean value of the relative index above, and then the weight of each index determined according to the CV method is:

$w_{i}=\frac{V_{i}}{\sum_{i=1}^{n} V_{i}}$ $i=1,2,\cdots,n$ (2)

Here, $0\leq w_{i}\leq1$, the higher the$w_{i}$ value is, the more important the index is in measuring farmland financial innovation. Table2 presents the weight of the third-level indexes in Table1.

Table2. The weight of third-level indexes of farmland financial innovation

| Index | Weight |
| --- | --- |
| Number of agricultural financial institutions per 10,000 square kilometers | 0.0880 |
| Number of employees in agricultural financial institutions per 10,000 square kilometers | 0.2356 |
| Total agricultural financial assets per 10,000 square kilometers | 0.1887 |
| Number of agricultural financial institutions per 10,000 people | 0.0420 |
| Number of employees in agricultural financial institutions per 10,000 people | 0.1654 |
| Total agricultural financial assets per 10,000 people | 0.1164 |
| Per capita agricultural loans balance | 0.0906 |
| The proportion of per capita agricultural loans to per capita income | 0.0773 |

Considering that the indexes with different dimensions cannot be compared directly, the linear threshold method is used to normalize each index. The specific calculation formula of each adjusted index is as follows:

$D_{i}=w_{i}\times\frac{X_{i}-m_{i}}{M_{i}-m_{i}}$ (3)

$X_{i}$ is the actual value of $i$ the index of a province in a year, $M_{i}$is the maximum value of $i$ index, $m_{i}$is the minimum value of $i$ index. The value range of $D_{i}$ index is $[0,w_{i}]$. The degree of farmland financial innovation in a province can be expressed in the form of a dimension vector$V_{i}=[D_{1},D_{2}\cdots D{}_{n}]$. In general, the farmland financial innovation $FFI$ can be synthesized by the Euclidean space distance between the measured value and the ideal value of each province in each year. The calculation formula is as follows:

 (4)

*3.2. Measuring labor migration*

Different from the two-way flow of labor force between urban and rural areas in developed countries and most developing countries, China's labor migration is a one-way flow of rural population to urban areas. This special one-way population migration model provides us with the possibility to calculate the flow of urban and rural labor forces. We can use the difference in the number of rural populations in the adjacent years to estimate the number of labor migration in that year. To improve the accuracy of the estimation, it is also necessary to consider the impact of natural population changes, such as birth rate and death rate, into the estimation formula. The rural death population who did not participate in the migration should be subtracted, while the newborn population who participated in the migration should be added. Fortunately, the existing statistical data can provide complete panel data of rural population, birth rate, and mortality rate of each province since 2005. On this basis, the labor migration can be calculated. The specific estimation formula is as follows: (1) The number of labor migration in a province in a certain year equals the number of rural populations in the province in the previous year minus the number of rural populations in the province in that year and the number of rural deaths in the province in that year, then add the number of rural newborn population in that year. (2) The number of rural death population in a province in a certain year equals the number of rural populations in the province in that year and multiply by the rural population death rate of the province in that year. (3) The number of rural-born population in a province in a certain year equals the number of rural populations in the province in that year and multiply by the birth rate of the rural population in that year.

3. **Estimation strategy**

This section illustrates the econometric specification, variable definitions, and data used in our paper.

*4.1. econometric specifications and variable definitions*

To verify the correlation between farmland financial innovation and the urban-rural income gap, we employ the following baseline multivariate econometric specification:

$URIG_{i,t}=\alpha_{0}+\alpha_{1}Fin_{i,t}+\alpha_{2}ISS_{i.t}+\alpha_{3}ER_{i.t}+\alpha_{4}TR_{i.t}+\alpha_{5}RT_{i.t}+\alpha_{6}Trans_{i,t}+\mu_{i}+\varepsilon_{i,t}$（5）

Where Eq.(5), The subscript $i$ represents a province(city) in China and the period is represented by $t$. To separately control province-specific heterogeneous characteristics, the individual fixed effect$\mu_{i}$ is included and$\varepsilon_{i,t}$ is the residual sum of squares. The dependent variable *URIG* represents the urban-rural income gap, measured by the income ratio between urban and rural areas. The core independent variable $Fin$ denotes farmland financial innovation. The mediation variable $Trans$ proxies for labor migration and the variable $ISS$ proxies for industrial structure upgrading. In addition, this paper also includes several control variables, namely: (1) rural Engel coefficient ($ER$); (2) per capita rural fixed telephone number ($RT$); (3) degree of dependence on foreign trade (*TR*). Table 3 summarizes the definitions and constructions of the variables used in the regressions. The coefficient $\alpha_{1}$ illustrates the effect of farmland financial innovation on the urban-rural income gap. The sign $\alpha_{1}$ is unclear based on our theoretical analysis.

Table 3. Variable definition and constructions

| Variable | Variable definition | Constructions |
| --- | --- | --- |
| *FIN* | farmland financial innovation | see above |
| *ISS* | industrial structure | The ratio of production value of  tertiary industry over total GDP |
| *URIG* | urban-rural income gap | The ratio of per capita disposable income of urban residents over per capita net income of rural residents |
| *TR* | degree of dependence on foreign trade | The ratio of the total export-import volume  over GDP |
| *ER* | rural Engel coefficient | The ratio of expenditure on necessities for rural residents over total expenditure |
| *RT* | rural per capita fixed telephone number | Number of telephones per 10000 rural residents |
| *Trans* | labor migration | see above |
| *UR* | urbanization rate | The ratio of urban resident population over the total population |

*4.2. Date and descriptive statistics*

We select 30 provinces (cities) in China (Tibet Province is not included) as the research samples. All raw data are collected at an annual frequency from 2006 to 2018. The data on these provinces are retrieved from China Statistical Yearbook, Statistical Yearbook of each province, Almanac of China's finance and banking, and China Urban-Rural Construction Statistical Yearbook. Given that a differential processing for the adjacent year is carried out in measuring the annual labor migration of each province, one year of data will be lost in the time series. Therefore, we finically collect panel data of 30 provinces from 2006 to 2017 in our regression model, with a total of 360 observations. Table 4 shows the descriptive statistics of each concerned variable. It can be seen from the Table that the minimum value of farmland financial innovation is 0.004 and the maximum value is 0.742, indicating that the farmland financial innovation varies greatly in the province

Table 4. Summary statistics for main variables.

| Variable | Mean | Std.Dev. | Min | P25 | Median | P75 | Max |
| --- | --- | --- | --- | --- | --- | --- | --- |
| *URIG* | 2.857 | 0.544 | 1.845 | 2.444 | 2.766 | 3.141 | 4.594 |
| *fin* | 0.081 | 0.109 | 0.004 | 0.032 | 0.051 | 0.078 | 0.742 |
| *iss* | 0.428 | 0.1 | 0.256 | 0.363 | 0.406 | 0.468 | 0.806 |
| *er* | 0.382 | 0.068 | 0.247 | 0.33 | 0.377 | 0.429 | 0.56 |
| *tr* | 0.309 | 0.374 | 0.017 | 0.091 | 0.14 | 0.357 | 1.721 |
| *rt* | 0.131 | 0.115 | 0.008 | 0.065 | 0.100 | 0.151 | 0.789 |
| *trans* | 4.53 | 1.37 | -1.833 | 3.763 | 4.953 | 5.496 | 6.505 |
| *gdp* | 9.409 | 0.944 | 6.475 | 8.889 | 9.529 | 10.046 | 11.404 |

Notes: The Table shows the descriptive statistics of the main variables in this research.

**5. Empirical results**

In this section, we conduct regression analysis based on Eq. (5) to investigate the impact of farmland financial innovation on the urban-rural income gap. Besides, we examine the possible mechanisms through which farmland financial innovation affects the urban-rural income gap. Finally, we perform robustness tests by controlling macroeconomic factors, increasing control variables, and changing model settings.

*5.1. Baseline analysis*

The Hausman test is employed to determine whether the random or fixed effect regression should be applied in the study. The result shows that the Chi-square statistic is 71.46, implying that the fixed effect model should be applied. Table 5 reports the baseline estimation results based on Eq. (5). To enhance the credibility of the estimation results, the control variables are gradually added to the baseline regression model. As shown in Column (1) of the Table, the coefficient of farmland financial innovation ($Fin$) is -4.131, which is significant at the 5% level. This result is still robust after gradually adding the control variables. As such, hypotheses 1-1 we presented earlier can be verified.

In addition, we also find that the industrial structure upgrading ($ISS$) can significantly bridge the urban-rural income gap ($URIR$). Although labor migration ($Trans$) has a negative impact on the urban-rural resident income gap ($URIR$), the coefficient is only -0.005 and its t-statistic is very small, failing to pass the significance level test. The potential transmission channels through which farmland financial innovation impacts the urban-rural income gap still require further examination.

Table 5. Results of the baseline regression.

|  | (1) | (2) | (3) | (4) | (5) | (6) |
| --- | --- | --- | --- | --- | --- | --- |
|  | *URIG* | *URIG* | *URIG* | *URIG* | *URIG* | *URIG* |
| *fin* | -4.131^**^ | -2.596 | -0.165 | -1.991 | -2.139^*^ | -2.105^***^ |
|  | (-2.09) | (-1.25) | (-0.17) | (-1.70) | (-1.96) | (-4.01) |
| *iss* |  | -2.044^**^ | -0.282 | -0.434 | -0.510 | -0.531^*^ |
|  |  | (-2.46) | (-0.66) | (-1.00) | (-1.19) | (-1.72) |
| *er* |  |  | 4.149^***^ | 3.826^***^ | 3.928^***^ | 3.941^***^ |
|  |  |  | (9.42) | (8.88) | (8.80) | (14.71) |
| *tr* |  |  |  | -0.712^**^ | -0.722^**^ | -0.721^***^ |
|  |  |  |  | (-2.52) | (-2.55) | (-5.88) |
| *rt* |  |  |  |  | -0.417 | -0.419 |
|  |  |  |  |  | (-0.98) | (-1.42) |
| *trans* |  |  |  |  |  | -0.005 |
|  |  |  |  |  |  | (-0.26) |
| *_cons* | 3.192^***^ | 3.944^***^ | 1.407^***^ | 1.964^***^ | 2.027^***^ | 2.053^***^ |
|  | (19.91) | (16.62) | (5.89) | (6.60) | (6.79) | (8.81) |
| *IE* | YES | YES | YES | YES | YES | YES |
| *Adj*-R^2^ | 0.219 | 0.282 | 0.598 | 0.635 | 0.638 | 0.638 |
| *N* | 360 | 360 | 360 | 360 | 360 | 360 |

Notes: ^*^, ^**^ and ^***^ indicate significance at 10%, 5%, and 1% confidence level respectively. The numbers in the parenthesis are corresponding t-values. IE means individual effect.

*5.2. Exploring the underlying mechanisms*

*5.2.1. Labor migration as the Channel*

The baseline regression model has confirmed that there is a negative correlation between farmland financial innovation and the urban-rural income gap. However, how does farmland financial innovation affect urban-rural residents' income ratio, in other words, what are the potential transmission channels in this relationship? Based on the previous theoretical analyses in the indirect effect hypothesis, farmland financial innovation can narrow the urban-rural income gap by eliminating farmer-land attachment, promoting the permanent migration of the labor force, and upgrading the industrial structure. We, therefore, have two potential mediate variables, namely, labor migration and industrial structure upgrading. Referring to the mediation effect test procedure of Baron et al.(1986) [34], we examine two potential transmission channels in this section and construct the equations as follows.

$Trans_{i,t}/ISS_{i,t}=\beta_{0}+\beta_{1}Fin_{i,t}+\beta Controls_{i,t}+u_{i}+\varepsilon_{i,t}$ （6）

$URIR_{i,t}=\gamma_{0}+\gamma_{1}Fin_{i,t}+\gamma_{2}Trans_{i,t}/ISS_{i,t}+\gamma Controls_{i,t}+u_{i}+\varepsilon_{i,t}$ （7）

The corresponding results are reported in Table 6 Column (1) investigates the effect of $Fin$ the mediate variable $Trans$. The coefficient $Fin$ is 6.271, significantly positive at the 5% level, which indicates that farmland financial innovation will accelerate the flow of labor force between urban and rural areas. In Column (2), the independent variable $Fin$ and the mediate variable $Trans$ are included to test the joint effects on the urban-rural income gap ($URIR$). The coefficient $Fin$ is significantly negative at the 10% level, and the coefficient $Trans$ is negative, which is consistent with our theoretical expectation. However, Column (2) further shows that the coefficient fails to pass the significance test. So the mediation impact should be further validated using the Sobel Test (Baron et al., 1986). The results of the Soble test shows that the Z-statistic value is -2, which is significant at the level of 5%. The partial mediating effect is verified and the mediation effect accounts for nearly 40%. Thus, this two-step analysis supports the interpretation that labor migration works as the mechanism behind farmland financial innovation and urban-rural income gap in China, that is, hypothesis 2 is verified.

*5.2.2. Industrial Structure Upgrading as the Channel*

To test whether industrial structure upgrading works as another channel through which farmland financial innovation affects urban-rural residents' income ratio, we first provide empirical evidence to show that $Fin$ does promote industrial upgrading. The corresponding results are shown in Column (3) of Table 6. More specifically, the coefficient of $Fin$ is 0.389, which is significantly positive at the level of 1%. Then we perform the second step test on Eq.(7), investigating the impact of the independent variable ($Fin$) on mediate variable ($ISS$). Both independent variable ($Fin$) and mediate variable ($ISS$) are included in the estimation. From Column (4) of Table 6, the coefficient of $Fin$ is significantly positive at the level of 1%, and the coefficient of$ISS$ is significantly negative at the level of 10%, indicating that industrial structure upgrading bridges the urban-rural residents' income gap in China. Here we also carry out the Soble test as above and the result shows that the Z-statistic is 3.908, which is significant at the level of 1%, signifying that the partial mediation effect is tenable. Thus, this two-step analysis supports the interpretation that the industrial structure upgrading works as the mechanism behind farmland financial innovation and urban-rural income gap in China, that is, hypothesis 3 is tenable.

Table 6. Results of the transmission channels.

|  | (1) | (2) | (3) | (4) |
| --- | --- | --- | --- | --- |
|  | *trans* | *URIG* | *iss* | *URIG* |
| *fin* | 6.271^**^ | -2.105^*^ | 0.389^***^ | -2.105^***^ |
|  | (2.10) | (-1.98) | (4.23) | (-4.01) |
| *iss* | -3.824 | -0.531 |  | -0.531^*^ |
|  | (-1.67) | (-1.25) |  | (-1.72) |
| *er* | 2.344^*^ | 3.941^***^ | -0.207^***^ | 3.941^***^ |
|  | (1.90) | (8.92) | (-4.42) | (14.71) |
| *tr* | 0.175 | -0.721^**^ | -0.035 | -0.721^***^ |
|  | (0.49) | (-2.55) | (-1.57) | (-5.88) |
| *rt* | -0.361 | -0.419 | -0.172^***^ | -0.419 |
|  | (-0.14) | (-0.97) | (-3.31) | (-1.42) |
| *trans* |  | -0.005 | -0.017^***^ | -0.005 |
|  |  | (-0.16) | (-4.79) | (-0.26) |
| _cons | 4.758^***^ | 2.053^***^ | 0.587^***^ | 2.053^***^ |
|  | (4.83) | (5.62) | (22.23) | (8.81) |
| *IE* | YES | YES | YES | YES |
| *Adj*-R^2^ | 0.145 | 0.638 | 0.473 | 0.638 |
| *N* | 360 | 360 | 360 | 360 |

Notes: ^*^, ^**^ and ^***^ indicate significance at 10%, 5%, and 1% confidence level respectively. The numbers in the parenthesis are corresponding t-values. IE means individual effect.

*5.3. Robustness Test*

To verify the reliability of our empirical findings, we conduct a battery of sensitivity tests. We re-launching a series of auxiliary verifications from multiple dimensions including replacing of regression method, and controlling more potential omitted variables, which provide further support for our main finding. We now turn to explain them in the following subsections.

*5.3.1. Controlling Macroeconomic Factors*

Given that controlling time fixed effect may lead to multicollinearity problems, referring to Pastor and Veronesi (2013), we add macroeconomic variables including economic development (*GDP* growth rate) and monetary policy (*M2* year-on-year growth rate) respectively into the baseline model (1) and then observe whether the impact of farmland financial innovation on urban-rural income gap changes significantly after controlling macroeconomic trend. Columns (1) to 2 of Table 8 report the estimation results after adding *GDP* growth rate and *M2* growth rate respectively. The coefficient of $Fin$ is still negative and significant at the 1% level, and the coefficients of the control variables also did not change significantly, which further verifies our previous conclusions.

Table 7. Robustness test: Controlling macroeconomic factors

| *variables* | (1) | (2) |
| --- | --- | --- |
|  | *URIG* | *URIG* |
| *fin* | -1.411^***^ | -1.423^***^ |
|  | (-2.72) | (-2.62) |
| *iss* | -0.397 | -0.357 |
|  | (-1.34) | (-1.17) |
| *er* | 3.084^***^ | 3.593^***^ |
|  | (10.27) | (12.98) |
| *tr* | -0.768^***^ | -0.584^***^ |
|  | (-6.52) | (-4.67) |
| *rt* | -0.506^*^ | -0.480^*^ |
|  | (-1.79) | (-1.66) |
| *trans* | -0.015 | -0.012 |
|  | (-0.74) | (-0.59) |
| *gdp* | 3.576^***^ |  |
|  | (5.49) |  |
| *M2* |  | 1.056^***^ |
|  |  | (3.92) |
| *_cons* | 2.009^***^ | 1.888^***^ |
|  | (8.99) | (8.14) |
| *IE* | YES | YES |
| *Adj*-R^2^ | 0.669 | 0.654 |
| N | 360 | 360 |

Notes: ^*^, ^**^ and ^***^ indicate significance at 10%, 5%, and 1% confidence level respectively. The numbers in the parenthesis are corresponding t-values. IE means individual effect.

*5.3.2. Addressing the potential endogeneity issue*

The above analysis of the paper may not have full control over the urban and rural development characteristics of each province, which may lead to the problem of missing variables. To eliminate the potential impact of endogeneity problems on the panel regression model, we continue to add rural per capita education years ($edu$) and urbanization rate ($UR$) as control variables given the importance of human capital and economic development level in the empirical study of urban and rural residents income distribution. Table 8 reports the regression results when gradually adding control variables.

Column (8) of Table 8 presents the result of the regressions including $edu$, $UR$ other concerned control variables. Compared with Column (6), we can find that although the coefficient of $Fin$ shows a slight decrease, the sign and significance of $Fin$ show no obvious change. As such, hypotheses 1-1 we presented earlier can be verified again. Column (7) of Table 8 further shows that the coefficient of $edu$ is significantly negative at a 1% level, implying that improving farmers' education level is an essential part of narrowing the income gap between urban and rural areas. Column (8) of Table 8 further shows that the coefficient of $UR$ is also significantly negative at the 1% level. This confirms that urbanization is part of an inclusive and healthy economic growth strategy, which is in line with the theoretical expectation.

Table 8. Robustness test: Increasing possible missing variables

| *variables* | (1) | (2) | (3) | (4) | (5) | (6) | (7) | (8) |
| --- | --- | --- | --- | --- | --- | --- | --- | --- |
|  | *URIG* | *URIG* | *URIG* | *URIG* | *URIG* | *URIG* | *URIG* | *URIG* |
| *fin* | -4.131^**^ | -2.596 | -0.165 | -1.991 | -2.139^*^ | -2.105^***^ | -0.988^*^ | -1.159^**^ |
|  | (-2.09) | (-1.25) | (-0.17) | (-1.70) | (-1.96) | (-4.01) | (-1.82) | (-2.28) |
| *iss* |  | -2.044^**^ | -0.282 | -0.434 | -0.510 | -0.531^*^ | -0.454 | -0.219 |
|  |  | (-2.46) | (-0.66) | (-1.00) | (-1.19) | (-1.72) | (-1.54) | (-0.79) |
| *er* |  |  | 4.149^***^ | 3.826^***^ | 3.928^***^ | 3.941^***^ | 3.075^***^ | 1.434^***^ |
|  |  |  | (9.42) | (8.88) | (8.80) | (14.71) | (10.23) | (3.88) |
| *tr* |  |  |  | -0.712^**^ | -0.722^**^ | -0.721^***^ | -0.673^***^ | -0.525^***^ |
|  |  |  |  | (-2.52) | (-2.55) | (-5.88) | (-5.72) | (-4.68) |
| *rt* |  |  |  |  | -0.417 | -0.419 | -0.523^*^ | -0.578^**^ |
|  |  |  |  |  | (-0.98) | (-1.42) | (-1.85) | (-2.19) |
| *trans* |  |  |  |  |  | -0.005 | -0.012 | 0.014 |
|  |  |  |  |  |  | (-0.26) | (-0.61) | (0.76) |
| *edu* |  |  |  |  |  |  | -0.200^***^ | -0.041 |
|  |  |  |  |  |  |  | (-5.52) | (-0.99) |
| *ur* |  |  |  |  |  |  |  | -3.406^***^ |
|  |  |  |  |  |  |  |  | (-6.86) |
| *_cons* | 3.192^***^ | 3.944^***^ | 1.407^***^ | 1.964^***^ | 2.027^***^ | 2.053^***^ | 4.046^***^ | 4.850^***^ |
|  | (19.91) | (16.62) | (5.89) | (6.60) | (6.79) | (8.81) | (9.54) | (11.72) |
| *IE* | YES | YES | YES | YES | YES | YES | YES | YES |
| *Adj*-R^2^ | 0.219 | 0.282 | 0.598 | 0.635 | 0.638 | 0.638 | 0.669 | 0.711 |
| N | 360 | 360 | 360 | 360 | 360 | 360 | 360 | 360 |

Notes: ^*^, ^**^ and ^***^ indicate significance at 10%, 5%, and 1% confidence level respectively. The numbers in the parenthesis are corresponding t-values. IE means individual effect.

*5.3.3.* *Alternative metrology method*

In this section, to deal with potential heteroscedasticity and autocorrelation problems, *MLE* estimation methods are used for the robustness tests. Maximum likelihood estimation (*MLE*) is not sensitive to the standardization of parameters and models. Although the *MLE* estimator is asymptotically distributed, it is still more effective and has less estimation bias than moment estimation in relatively small samples.

The corresponding regression results are shown in Columns (1) to (6) of Table 7. With this model specification, a 1% increase $Fin$ is significantly associated with a 1.171% decrease $URIG$ after all control variables are included, which further strengthens our previous findings.

Table 9. Robustness test: Alternative metrology method

|  | (1) | (2) | (3) | (4) | (5) | (6) |
| --- | --- | --- | --- | --- | --- | --- |
|  | *URIG* | *URIG* | *URIG* | *URIG* | *URIG* | *URIG* |
| *fin* | -3.603^***^ | -2.222^***^ | -0.332 | -1.207^***^ | -1.211^***^ | -1.171^***^ |
|  | (-8.95) | (-4.71) | (-0.89) | (-2.99) | (-2.99) | (-2.87) |
| *iss* |  | -1.944^***^ | -0.268 | -0.268 | -0.280 | -0.332 |
|  |  | (-5.22) | (-0.90) | (-0.94) | (-0.97) | (-1.11) |
| *er* |  |  | 4.082^***^ | 4.062^***^ | 4.086^***^ | 4.108^***^ |
|  |  |  | (16.47) | (16.95) | (16.04) | (16.00) |
| *tr* |  |  |  | -0.523^***^ | -0.519^***^ | -0.526^***^ |
|  |  |  |  | (-5.50) | (-5.37) | (-5.43) |
| *rt* |  |  |  |  | -0.075 | -0.086 |
|  |  |  |  |  | (-0.28) | (-0.32) |
| *trans* |  |  |  |  |  | -0.013 |
|  |  |  |  |  |  | (-0.65) |
| *_cons* | 3.149^***^ | 3.870^***^ | 1.441^***^ | 1.681^***^ | 1.686^***^ | 1.757^***^ |
|  | (32.99) | (22.90) | (7.28) | (8.42) | (8.39) | (7.69) |
| *sigma_u* | 0.486^***^ | 0.502^***^ | 0.425^***^ | 0.414^***^ | 0.417^***^ | 0.416^***^ |
|  | (7.25) | (7.32) | (7.59) | (7.17) | (7.03) | (7.07) |
| *sigma_e* | 0.258^***^ | 0.247^***^ | 0.185^***^ | 0.177^***^ | 0.177^***^ | 0.177^***^ |
|  | (25.59) | (25.60) | (25.68) | (25.54) | (25.49) | (25.50) |
| *N* | 360 | 360 | 360 | 360 | 360 | 360 |

Notes: ^*^, ^**^ and ^***^ indicate significance at 10%, 5%, and 1% confidence level respectively. The numbers in the parenthesis are corresponding t-values.

**5.4. Further Analysis: The role of the heterogeneity in the macroeconomic cycle**

The above research takes the province-level panel date from 2006 to 2017 as the sample and provides empirical verification of how farmland financial innovation narrows the income gap between urban and rural areas. As a matter of fact, during the sample period, the macro-economic environment of China has changed greatly. Especially after 2014, China's economy gradually changed from a high speed of growth to a medium-to-high speed of growth. Along with this comes a problem that is whether the impact of farmland financial innovation on the rural and urban income distribution appears to be heterogeneous due to economic fluctuations, thus requiring further group tests on economic expansion and economic depression period. At present, downward pressure on China's economy has continued to mount, and we have faced an array of interwoven difficulties and challenges. In this background, the role of farmland financial innovation on the urban-rural income gap being weakened or more prominent is worth further study.

We now examine whether the impact of farmland financial innovation on the urban-rural income gap appears to be heterogeneous in different stages of the economic cycle. We adopt Hodrick-Prescott (*HP*) filter to measure the macroeconomic cycle. The Hodrick-Prescott (*HP*) filter refers to a data-smoothing technique. The *HP* filter is commonly applied during the analysis to remove short-term fluctuations associated with the business cycle. To overcome the defect of short sample data, we use Eviews 9.0 to process the national economic growth rate from 2003 to 2018 by *HP* filter. If the extracted cyclical component is greater than zero, it is recorded as *cycle* = 1, which denotes the period of economic expansion. Otherwise, it is recorded as *cycle* = 0, which denotes the period of economic depression. Table 10 reports the regression results of the baseline model for the period of economic expansion (5 years) and the period of economic depression (10 years) respectively. Compared with the regression results of the baseline model, the coefficient sign $Fin$ remains negative during the period of economic expansion, but not significant. In contrast, in the sub-sample regression of the economic depression period, the impact of $Fin$ on urban-rural income gap remains significant at a 1% level and even more powerful. Our findings show that especially in the economic downturn, the role of farmland financial innovation in narrowing the income gap between urban and rural areas is more prominent.

The economic intuition behind this finding can be illustrated as follows. Usually, when the economy enters a recession stage, banks are very cautious about offering new loans, thus risk assessments can be more stringent. The financing situations of farmers in the capital market become more unfavorable; the contradiction between supply and demand of agricultural production and operation funds seems more acute, so the demand for farmland financial innovation could be more urgent. At the same time, the employment capacity of non-agricultural industries decreases enormously when the economy is in a downward stage, forcing farmers to increase their dependence on the land. In general, when farmers are trapped in troubles in increasing income by other ways during the downward period, the channel of farmland financial innovation to urban-rural income gap is more and more prominent.

Table 10. The heterogeneity test of the macroeconomic cycle

| *Variables* | Dependent variable: URIG |  |
| --- | --- | --- |
|  | Economic expansion period  (5 years) | Economic depression period  (10 years) |
| *fin* | -0.553 | -2.851^***^ |
|  | (-0.44) | (-3.12) |
| *iss* | -0.590 | -0.715^*^ |
|  | (-1.22) | (-1.81) |
| *er* | 4.361^***^ | 3.804^***^ |
|  | (7.59) | (8.27) |
| *tr* | -0.376 | -1.156^***^ |
|  | (-1.31) | (-4.90) |
| *rt* | -0.665^*^ | -0.255 |
|  | (-1.75) | (-0.50) |
| *trans* | -0.020 | -0.027 |
|  | (-0.61) | (-0.75) |
| *_cons* | 1.812^***^ | 2.432^***^ |
|  | (4.46) | (6.32) |
| *IE* | Yes | Yes |
| *Adj*-R^2^ | 0.729 | 0.615 |
| N | 150 | 210 |

Notes: ^*^, ^**^ and ^***^ indicate significance at 10%, 5%, and 1% confidence level respectively. The numbers in the parenthesis are corresponding t-values.

**6. Conclusions**

Given the dilemma of the urban-rural income gap in China explained by the neoclassical equilibrium theory, we attempt to provide a new theoretical explanation for the high-income gap between urban and rural areas in China. This paper selects data from 30 provinces (cities) in China over the period from 2003 to 2017 as a sample to investigate whether and how the degree of farmland financial innovation narrows the urban-rural income gap. Our results show that farmland financial innovation has a significant negative impact on the urban-rural income gap, which is more intense during the economic recession. The mediation effect provides evidence that farmland financial innovation can narrow the urban-rural income gap through eliminating farmer-land attachment, promoting the permanent migration of the labor force, and upgrading the industrial structure.

Our research has far-reaching policy implications. Firstly, China should promote various forms of farmland financial innovation, such as mortgage loan of a contracted land management right, shares of land use right, farmland trust, which can not only alleviate the contradiction of capital supply and demand in the development of "agriculture, rural areas and farmers", but also help to narrow the current high-income gap between urban and rural areas.

Secondly, policymakers must remove the administrative barriers for urban funds to the countryside to change the one-way flow of labor to the city. They also should strengthen the reform of the hukou, education, and social security systems, to promote the permanent migration of middle and low-income rural residents to cities.

Thirdly, it is necessary to establish a rural property rights transaction system, and select economically developed areas to try out a paid withdrawal system for the farmland contract management rights if conditions permit. Land contract rights should be separate from the hukou system, so those rural residents can migrate to the city and settle down without fear of losing their land.

**References**

1. Acemoglu,D.;Guerrieri,V. Capital deepening and non-balanced economic growth. Journal of Political Economy, 2008,3,467-498.
2. Aghion, P.; Bolton, P. Distribution and growth in models of imperfect capital markets. European Economic Review,1992,36,2-3,603-611.
3. Alain De Janvary;Emerick,K.;Marco Gonzalez-Navarro;Sadoulet,E. Delinking land rights from land use: certification and migration in Mexico. American Economic Review, 2015,10,3125-3149.
4. Baron, R.M.; Kenny, D.A. The moderator-mediator variable distinction in social psychological research: conceptual, strategic, and statistical considerations. Journal of Personality and Social Psychology, 1986,6, 1173-1182.
5. Baumol, W.J. Macroeconomics of unbalanced growth: the anatomy of urban crisis. American Economic Review, 1967,3,415-426.
6. Beck, T.; Levine, R.; Loayza, N. Finance and the sources of growth. Journal of Financial Economics, 2000,58,261-310.
7. Bhaduri,A.;Skarstein,R. Effective demand and the terms of trade in a dual economy: a Kaldorian perspective. Cambridge Journal Economics, 2003,4,583-595.
8. Bose, G. Agrarian efficiency wages in a dual economy. Journal of Development Economics, 1996,2,371-386.
9. Carrillo, B. Rural-urban migration in China: temporary migrants in search of permanent settlement. Journal of Multidisciplinary International Studies 2004,2,1-26.
10. Fisher, A.G.B. A note on tertiary production. Economic Journal 1952,248,820-834.
11. Fleisher, B.M.; Chen, J. The coast-non coast income gap, productivity, and regional economic policy in China. Journal of Comparative Economics, 1997,2,220-236.
12. Galor, O.; Zeira, J. Income distribution, and macroeconomics. Review of Economic Studies, 1993,1,35-52.
13. Ge D: Long H; Qiao W: Sun D; & Yang R. Effects of rural-urban migration on agricultural transformation: a case of yucheng city, china. Journal of Rural Studies, 2020.
14. Greenwood,J.;Jovanovic,B. Financial development, growth, and the distribution of income. Journal of Political Economy 1990,5,1076-1107.
15. Guo, J.X. Human capital, the birth rate and the narrowing of the urban-rural income gap. Social Sciences in China 2005,3,28-37.
16. Henderson, J.V.; Wang, H.G. Urbanization and city growth: the role of institutions. Regional Science & Urban Economics 2007,3,283-313.
17. Honohan, P. Perverse effects of an external ratings-related capital adequacy system. Economic Notes 2001, 3,359-372.
18. Jalilian, H.; Kirkpatrick, C. [Financial development and poverty reduction in developing countries](https://xueshu.baidu.com/usercenter/paper/show?paperid=2c704ce2f66c1a5eceef70b885d0cab7). International Journal of Finance & Economics 2002,2,97-108.
19. Jeanneney,S.G.;Kpodar,K. Financial development and poverty reduction: can there be a benefit without a cost? Journal of Development Studies 2011,1,143-163.
20. Kuznets, S. Economic growth and income inequality. American Economic Review 1955, 1,1-28.
21. Li, H.Z.; Zahniser, S. The determinants of temporary rural-to-urban migration in China. Urban Studies 2002,12,2219-2235.
22. Lin, Y.F.; Liu, P.L. Chinese development strategy and economic convergence. Economic Research Journal 2003,3,19-25. (In Chinese)
23. Lu, M; Chen, Z. Urban-biased economic policies and Urban-rural inequality. Economic Research Journal 2004,6,50-58. (In Chinese)
24. Lucas, R.E. Life earnings and rural-urban migration. Journal of Political Economy 2004,1,29-59.
25. Mullan,M.;Grosjean,P.;Kontoleon,A. Land tenure arrangements and rural-Urban migration in China. Word development, 2011,1,123-133.
26. Nelson, R.R.; Phelps, E.S. Investment in humans, technological diffusion, and economic growth. American Economic Review 1966,1/2,69-75.
27. North, D.C. Structure and change in economic history. W.W.Norton&Company New York,1981.
28. Pastor, L.; Veronesi, P. Political uncertainty and risk premia. Journal of Financial Economics 2013,3,520-545.
29. Pu, J. Liberating land -- a new round of land trust reform. China CITIC Press Beijing,2014.
30. Rin, M.D.; Hellmann, T. Banks as catalysts for industrialization. Journal of Financial Intermediation 2002,4,366-397.
31. Shi, X.Z.; Sicular, T.; Zhao, Y.H. Analyzing urban-rural income inequality in China. International Symposium on Equity and Justice in Transitional China; Beijing,2002, July 11-12.
32. Sicular,T.;Yue,X.M.;Gustafsson,B.;Li,S. The urban-rural income gap and inequality in China. Review of Income and Wealth 2007,1,93-126.
33. Su, C. W., Liu, T. Y., Chang, H. L., & Jiang, X. Z. . Is urbanization narrowing the urban-rural income gap? a cross-regional study of china. Habitat International, 2015, 48, 79-86.
34. Valsecchi, M. Land property rights and international migration: evidence from Mexico. Journal of Development Economics 2014,9,276-290.
35. Wan, H.Y.; Li, S. The effects of household registration system discrimination on urban-rural income inequality in China. Economic Research Journal 2013,9,43-55. (In Chinese)
36. Wang, L., & Zhang, M.. Exploring the impact of narrowing urban-rural income gap on carbon emission reduction and pollution control. PLOS ONE 2021, 16.
37. Wang, S. X., & Benjamin, F. Y. . Labor mobility barriers and rural-urban migration in transitional china. China Economic Review,2019, 53.
38. Wang X, Shao S, Li L. Agricultural inputs, urbanization, and urban-rural income disparity: Evidence from China. China Economic Review. 2019; 55:67–84
39. Xie, D.S. Rural land transfer rights, labor migration, and urban-rural inequality. Economic Issues China 2014,1,49-59.
