# Supplementary material for: Is farmland financial innovation narrowing the urban-rural income gap? A cross-regional study of China
Source: PLoS One. 2022 Jun 3;17(6):e0269503. doi: 10.1371/journal.pone.0269503 (PMC9165774; doi:10.1371/journal.pone.0269503)
Supplement: Attachment — Submitted filename: Response to Reviewers.docx [file pone.0269503.s003.docx]

**Responses to the Comments of the paper entitled “Is farmland financial innovation narrowing the urban-rural income gap? A cross-regional study of China” submitted to PLOS ONE.**

**(Manuscript Number: PONE-D-21-41037)**

**We bold in Blue our responses to the referee’s suggestions and comments.**

**We would like to thank the Guest Editor and three referees for valuable and guiding comments. We really appreciate your efforts in reviewing our study and your suggestions to improve the paper. We have paid high attention to each of your comments and tried to do our best to revise this paper.**

**The current manuscript has been copy-edited by American Journal Expert (AJE). We believe the flow of the paper has been greatly improved and grammatical errors have been avoided.**

**We highlight all the changes in red in file labeled ‘Revised Manuscript with Track Changes’.**

**Response to Guest Editor’s comments:**

1. Please ensure that your manuscript meets PLOS ONE's style requirements.

**We carefully read the PLOS ONE style templates and edit our manuscript according to the guidelines for main body, affiliations and file naming to ensure our revised manuscript meets PLOS ONE's style requirements.**

2. Please review your reference list to ensure that it is complete and correct. If you have cited papers that have been retracted, please include the rationale for doing so in the manuscript text, or remove these references and replace them with relevant current references. Any changes to the reference list should be mentioned in the rebuttal letter that accompanies your revised manuscript. If you need to cite a retracted article, indicate the article’s retracted status in the References list and also include a citation and full reference for the retraction notice.

**In the revised draft, we delete the references with weak relevance to the topic of the article, and supplement the references that can support our findings. We make carefully check of the reference list to ensure that it is complete and correct.**

**The deleted references are as follows:**

Bhaduri,A.;Skarstein,R. Effective demand and the terms of trade in a dual

economy:a Kaldorian perspective. Cambridge Journal Economics 2003,4,583-595.

Bose,G. Agrarian efficiency wages in a dual economy. Journal of

Development Economics 1996,2,371-386.

Fleisher,B.M.;Chen,J. The coast-noncoast income gap, productivity, and

regional economic policy in China. Journal of Comparative Economics 1997,2,220-236.

Guo,J.X. Human capital, the birth rate and the narrowing of urban rural

income gap. Social Sciences in China 2005,3,28-37.

Lin,Y.F.;Liu,P.L. Chinese development strategy and economic convergence. Economic Research Journal 2003,3,19-25.(In Chinese)

Lu, M; Chen, Z. Urban-biased economic policies and Urban-rural inequality. Economic Research Journal 2004,6,50-58.(In Chinese)

Nelson, R. R.; Phelps, E.S. Investment in humans, technological diffusion, and economic growth. American Economic Review 1966,1/2,69-75.

North, D. C. Structure and change in economic history. W.W. Norton & Company New York,1981.

Pu, J. Liberating land -- a new round of land trust reform. China CITIC Press Beijing,2014.

Shi, X. Z.; Sicular, T.; Zhao, Y.H. Analyzing urban rural income inequality in China. International Symposium on Equity and Justice in Transitional

China; Beijing,2002,July 11-12.

**The added references are as follows:**

Chan K W.The household registration system and migrant labor in China

Notes on a debate[J]. Population and development review, 2010, 36(2):

357-364, <https://doi.org/10.1111/j.1728-4457.2010.00333.x>.

Zhou S, Cheung M. Hukou system effects on migrant children’s education inChina: Learning from past disparities[J]. International social work, 2017, 60(6): 1327-1342, <https://doi.org/10.1177/0020872817725134>.

Song Y.Cost-benefit analysis of the Hukou reform: Simulation evidence

from a theoretical labor market model[J]. China Economic Quarterly

International, 2021, 1(2): 109-119, <https://doi.org/10.1016/j.ceqi.2021.03.001>.

Banerjee AV, Duflo E. Inequality and Growth : What Can the Date Say?

Journal of Economic Growth. 2003;8(3):267-299, <https://doi.org/10.1023/A:1026205114860>.

De Haan J, Sturm J E. Finance and income inequality: A review and new evidence[J]. European Journal of Political Economy, 2017, 50: 171-195,

<https://doi.org/10.1016/j.ejpoleco.2017.04.007>.

3. We note that the grant information you provided in the ‘Funding Information’ and ‘Financial Disclosure’ sections do not match. When you resubmit, please ensure that you provide the correct grant numbers for the awards you received for your study in the ‘Funding Information’ section.

**We are sorry for omitting to declare funding information in ‘Financial Disclosure’ section and we have checked our grant number and ensured that we provided the correct grant number in the ‘Funding Information’ section.**

**“Funding Information: This work was supported by the National Social Science Foundation of China (19BJL104).”**

4. PLOS requires an ORCID iD for the corresponding author in Editorial Manager on papers submitted after December 6th, 2016. Please ensure that you have an ORCID iD and that it is validated in Editorial Manager.

**Thank you for reminding. I have updated my information and filled in**

**my ORCID ID in the ‘Personal Information’ field.**

**Response to Reviewer #1:**

**Thank you for your careful and thoughtful examination of our paper. Incorporating your comments, as well as those of the Editor, and the other referee, resulted in a greatly improved manuscript. We address each of your comments below.**

**A. General comments**
Please restructure (regroup) your work into an ordered manner (organized format, clearly showing sections such as (i) Abstract, (ii) Introduction, (iii) Methods and Materials (methodology), (iv) Results and Discussions, and (v) conclusions and Recommendations. Read on each section below for more details on what should be included in your restructured work.
**Response to the comments:**

**We have restructured our work into an ordered manner including five sections, namely (i) Introduction, (ii) Theoretical and Conceptual Framework, (iii) Materials and Methods, (iv) Empirical results and Discussions, and (v) Conclusions and policy implications. We have combined the original chapter 3 (Measuring farmland financial innovation and labor migration) and chapter 4 (Estimation strategy) into one chapter (Materials and Methods) to make the structure of the article more reasonable. We have made a detailed description of the revised content of each section in the following.**

1. Abstract section:
In your abstract; indicate the brief background of the study (even the sentence with two to three lines), purpose (objective), methodology (materials and methods) of the study, important findings (indicating the significance level – P values, eg significant at P < 0.05 level), conclusions, and recommendations (if any).

**Response to the comments:**

**Thanks so much for your suggestion and comment. We have reorganized our abstract according to your suggestion,** **indicating the brief background of the study, purpose, materials and methods, important findings, the significance level and policy implications. The corresponding revisions are as follows:**

“Over the past four decades, China's economy has experienced tremendous economic growth but also a widening urban-rural income gap. Given the dilemma of the urban-rural income gap in China explained by neoclassical equilibrium theory, this paper attempts to provide a new theoretical explanation for the large-income gap between urban and rural areas in China. We select data from 30 provinces(cities) in China over the period from 2006 to 2017 as a sample to investigate whether and how the degree of farmland financial innovation narrows the urban-rural income gap. The results show that the coefficient for farmland financial innovation is significantly negative at the 1% level, signifying that financial innovation can narrow the urban-rural income gap in China. The mediation effect test provides evidence that farmland financial innovation narrows the urban-rural income gap by promoting the permanent migration of the labor force and upgrading the industrial structure. Our results indicate that the government should promote various forms of farmland financial innovation, establish rural property rights transaction system and free farmers from deep farmer-land attachment to realize permanent labor migration.”

2.Introduction section:
In the introduction, please indicate the background of your study, statement of the problem (reasons for undertaking this study), related literature reviewed; and indicating your objective of the study (purpose of your study) as well as the significance of the study. You have all these in your paper but are mixed up, make it clear.

**Response to the comments:**
**We greatly appreciate your comments and suggestions.** **We have rearranged our introduction and clearly indicated the background of our study, reasons for undertaking this study, related literature reviewed, and the significance of the study. In the first and second paragraphs we point out the dilemma of current research. That’s why we attempt to provide a new theoretical explanation for the high-income gap between urban and rural areas in China. Then we clarify the existing problems of China's agricultural land financial system and these situations hinder income convergence between urban and rural areas. Then we summarize existing studies on rural land finance and urban-rural income into three main viewpoints. Finally, we emphasize the three marginal contributions of our paper. We believe that the quality of the revised version of the paper has been greatly improved.**

3. Materials and methods (methodology).
The methodology is mixed up with the introduction. Indicate it clearly, stating clearly how you conducted your study (describing the study area, sampling procedures and sample size studied, data collection methods (tools), and data analysis techniques (empirical or analytical models).

**Response to the comments:**

**Thank you for this valuable comment. We have combined the original section 3 (Measuring farmland financial innovation and labor migration) and section 4 (Estimation strategy) into one chapter (Materials and Methods) to make the structure of the article more reasonable as you suggested. In the reviesd version, section 3 (Materials and Methods) illustrate our study area, data sources (see in 3.3), data processing procedures (see in 3.2) and empirical models (see in 3.3).**

4.Indicate your theoretical/ conceptual framework:
Indicate it clearly, state the theory or conceptual framework which guides your research/ study.

**Response to the comments:**

**Thank you for this insightful suggestion. We have stated our theoretical framework in section 2. We conduct our studies based on three hypothesizes using the theories of new institutional economics, development economics and land economics comprehensively. These hypothesizes are as follows:**
“Hypothesis 1-1: The poverty reduction effect of rural land financial development is greater than the threshold effect, and farmland financial innovation is useful to narrow the urban-rural income gap.

Hypothesis 1-2: The threshold effect of rural land financial development is greater than the poverty reduction effect, and farmland financial innovation will expand the urban-rural income gap.

Hypothesis 2: Farmland financial innovation promotes the permanent migration of the rural labor force, and the flow of population reduces the income gap between urban and rural areas.

Hypothesis 3: Farmland financial innovation promotes the upgrading of industrial structure, and optimizing the industrial structure is useful in narrowing the urban-rural income gap.”

5. Results and discussions sections:
Please indicate and discuss the results obtained from your analyzed data, relating your results with other authors' results.

**Response to the comments:**

**Thanks so much for your suggestion and comment. In section 4 (Empirical results and Discussions), we add discussions about our results obtained from analyzed data and relate our findings with other authors based on our findings. The corresponding revisions are as follows:**

“As shown in Column (6) of the table, the coefficient of farmland financial innovation ($FFI$) is -2.105, which is significant at the 1% level after adding all the control variables. As such, Hypotheses 1-1 that we presented earlier can be verified. This result indicates that the poverty reduction effect of farmland financial innovation is greater than the threshold effect; in other words, farmland financial innovation can prominently narrow the urban-rural income gap. This finding is in line with the research of Jalilian and Kirkpatrick, Honohan, Jeanneney and Kpodar [21, 22, 25].”

“Thus, this two-step analysis supports the interpretation that labor migration works as the mechanism behind farmland financial innovation and the urban-rural income gap in China; that is, Hypotheses 2 is verified. This result is consistent with the research of Xie, who hold that incomplete land transfer rights restrict the asset attributes and liquidity of land, makes it difficult for migrants to settle permanently in the city [9].”

“Thus, this two-step analysis supports the interpretation that industrial structure upgrading works as the mechanism behind farmland financial innovation and the urban-rural income gap in China; that is, Hypotheses 3 is tenable. Financial innovation in agriculture has accelerated industrial upgrading, improving the efficiency of labor markets and factor allocation, thus expanding the ways for rural residents to obtain jobs and increasing their income substantially, which supports the research of Acemoglu and Guerrieri, Rin and Hellmann [30,31].”
6. Conclusion section: Your conclusions should adhere to or be based on your objectives.
**Response to the comments:**

**Thank you for reminding. We have enriched the conclusions to connect them with our objectives tightly.**

“This paper is thus motivated to address the following questions: Can farmland financial innovation narrow the urban-rural income gap? What are the potential transmission channels through which the farmland financial innovation narrows the urban-rural income gap? The unique land system and economic background make China a particularly interesting and suitable case for analyzing the above questions.”

**Theoretical analysis and empirical test of this paper confirm that farmland financial innovation can significantly narrow the income gap between urban and rural areas, which also means that the poverty reduction effect of rural finance development is greater than the threshold effect. The mediation effect test provides evidence that farmland financial innovation can narrow the urban-rural income gap through promoting the permanent migration of the labor force and upgrading the industrial structure. So, in the revised manuscript, the conclusions are more closely related to the objective.**

**B. Additional Comments**

1. Citation and referencing: Citations should be by a number like [3] and not by publication year ( Akiba, 2020]. Read the guidelines for citations and referencing.
**Response to the comments:**

**Thank you for reminding. We carefully read the formatting sample and readjust all citations of the paper according to the guidelines for citations and referencing.**

2. Grammarly check:
   I have tried to check some grammatical errors, please check the suggestions in the attachment.

**Response to the comments:**

**We are very sorry for the grammatical errors in this paper. Thank you so much for your careful suggestions in the attachment. We have paid high attention to those expressions that might be inappropriate, and have followed your suggestions to make careful revisions to language. The current manuscript has been copy-edited by American Journal Expert (AJE). Now, these mistakes have been dealt with in this version.**

3.Formatting: Consider formatting the font size and spacing for your headings and subheadings; for example the font size for heading 1 should be 18, heading 2 should be 16, etc. The spacing should be double spaced (Spacing of 2). Read the guidelines for font type and size. See suggestions in the attached file.
**Response to the comments:**

**Thank you for reminding! We carefully read the formatting sample in the attached file. In the revised manuscript, we have adjusted the format of the full text as required.**

4. Ethical considerations
Please indicate the ethical issues regarding the permission to conduct the research (data collection permission).

**Response to the comments:**

**Thank you for your advice. In this paper, we select 30 provinces (cities) in China (Tibet Province is not included) as the research sample. All raw data are collected at an annual frequency from 2006 to 2018. The sub-indicators of the financial farmland innovation index are manually collected from the China Finance Statistical Yearbook and China Rural Finance Statistical Yearbook. Other provincial control variables are retrieved from the statistical yearbook of provinces and cities and the China Urban-Rural Construction Statistical Yearbook. The GDP data are retrieved from the website http://www.stats.gov.cn/. All yearbooks and websites are open to the public and we have described the data processing in detail in Section 3.1.**

NOTE: Most of the details requested are in your paper, what you need is to rearrange them into an organized manner.

**Response to the comments:**

**Thanks very much for your suggestions and comments. We have rearranged them into an organized manner as you suggested above. Please see red area of the revised manuscript. We hope that our efforts have succeeded in allaying your concerns.**

**Response to Reviewer #2:**

**We would like to express our thanks for your time and effort in reviewing our paper. Thanks so much for your suggestions and for the helpful comments below. We have carefully revised the introduction, theoretical framework, materials and methods, empirical results and discussions of the paper, and we believe that the quality of the revised version of the paper has been greatly improved.**

1. The authors may shorten the length of the paper by giving important findings.

**Thanks very much for your suggestion and comment.**

**We have deleted the parts that are not closely related to the theme of the article (eg, original section 5.4: Further Analysis) and try to give more important findings. The number of words in the revised manuscript is 7556.**

**This paper is motivated to address the following questions: Can farmland financial innovation narrow the urban-rural income gap? What are the potential transmission channels that the farmland financial innovation narrows the urban-rural income gap? The unique land system and economic background make China a particularly interesting and suitable case for analyzing the above questions.**

**Based on our research objectives, we have the following major findings.**

**The results panel regression model in section 3.3 display that the coefficient for farmland financial innovation is significantly negative at the 1% level, indicating that farmland financial innovation can significantly narrow the income gap between urban and rural areas, which also means that the poverty reduction effect of rural finance development is greater than the threshold effect. The mediation effect test provides evidence that farmland financial innovation can narrow the urban-rural income gap through promoting the permanent migration of the labor force and upgrading the industrial structure. So, in the revised manuscript, the conclusions are more closely related to the objective.**

2. findings can be supported using the previous studies
**Response to the comments:**

**Thanks so much for your suggestion and comment. In section 4 (Empirical results and Discussions), we add discussions about our results obtained from analyzed data and relate our findings with other authors based on our findings. The corresponding revisions are as follows:**

“As shown in Column (6) of the table, the coefficient of farmland financial innovation ($FFI$) is -2.105, which is significant at the 1% level after adding all the control variables. As such, Hypotheses 1-1 that we presented earlier can be verified. This result indicates that the poverty reduction effect of farmland financial innovation is greater than the threshold effect; in other words, farmland financial innovation can prominently narrow the urban-rural income gap. This finding is in line with the research of Jalilian and Kirkpatrick, Honohan, Jeanneney and Kpodar [21, 22, 25].”

“Thus, this two-step analysis supports the interpretation that labor migration works as the mechanism behind farmland financial innovation and the urban-rural income gap in China; that is, Hypotheses 2 is verified. This result is consistent with the research of Xie, who hold that incomplete land transfer rights restrict the asset attributes and liquidity of land, makes it difficult for migrants to settle permanently in the city [9].”

“Thus, this two-step analysis supports the interpretation that industrial structure upgrading works as the mechanism behind farmland financial innovation and the urban-rural income gap in China; that is, Hypotheses 3 is tenable. Financial innovation in agriculture has accelerated industrial upgrading, improving the efficiency of labor markets and factor allocation, thus expanding the ways for rural residents to obtain jobs and increasing their income substantially, which supports the research of Acemoglu and Guerrieri, Rin and Hellmann [30,31].”

3.Manuscript can be edited according to author's guidelines

**Response to the comments:**

**We have restructured our work into an ordered manner including five sections, namely (i) Introduction, (ii) Theoretical and Conceptual Framework, (iii) Materials and Methods, (iv) Empirical results and Discussions, and (v) Conclusions and policy implications. In the revised manuscript, we have combined the original chapter 3 (Measuring farmland financial innovation and labor migration) and chapter 4 (Estimation strategy) into one chapter (Materials and Methods) to make the structure of the article more reasonable. We have carefully revised and improved each section. Please see red area of the revised manuscript.**

4. Authors may mention the data source as well for the different variables.

**Response to the comments:**

**Thank you for reminding! We have supplemented the data source of the different variable in section 3.3 of the revised manuscript.** **The corresponding revisions are as follows:**

“We select 30 provinces (cities) in China (Tibet Province is not included) as the research sample. All raw data are collected at an annual frequency from 2006 to 2018. The sub-indicators of the financial farmland innovation index are manually collected from the China Finance Statistical Yearbook and China Rural Finance Statistical Yearbook. Other provincial control variables are retrieved from the statistical yearbook of provinces and cities and the China Urban-Rural Construction Statistical Yearbook. The GDP data are retrieved from the website http://www.stats.gov.cn/. We have described the data processing in detail in Section 3.1.”

**Response to Reviewer #3:**

The paper addresses an important topic and the statistical analysis performed to meet the objectives are worth appreciation. However, English editing is recommended to improve the flow of the paper and avoid grammatical errors. Some of such errors are mentioned below but is not exhaustive. I am happy to accept the article after addressing the specific comments given below:

**Response to the comments:**

**Thank you for your careful and thoughtful examination of our paper. We are pleased to learn that you think we address an important topic and preform the statistical analysis adhering to our objectives. We highly value your suggestions and comments on this article and try our best to revise this paper.** **The current manuscript has been copy-edited by American Journal Expert (AJE).** **We believe the flow of the paper has been greatly improved and grammatical errors have been avoided. We address each of your comments below.**

1. The abstract may be re-written with language editing

**Response to the comments:**
**We thank you for your valuable comment. We have rewritten the abstract, indicating the brief background of the study, purpose, materials and methods, important findings, the significance level and policy implications. And the current manuscript has been copy-edited by American Journal Expert (AJE). The corresponding revisions are as follows:**

“Over the past four decades, China's economy has experienced tremendous economic growth but also a widening urban-rural income gap. Given the dilemma of the urban-rural income gap in China explained by neoclassical equilibrium theory, this paper attempts to provide a new theoretical explanation for the large-income gap between urban and rural areas in China. We select data from 30 provinces(cities) in China over the period from 2006 to 2017 as a sample to investigate whether and how the degree of farmland financial innovation narrows the urban-rural income gap. The results show that the coefficient for farmland financial innovation is significantly negative at the 1% level, signifying that financial innovation can narrow the urban-rural income gap in China. The mediation effect test provides evidence that farmland financial innovation narrows the urban-rural income gap by promoting the permanent migration of the labor force and upgrading the industrial structure. Our results indicate that the government should promote various forms of farmland financial innovation, establish rural property rights transaction system and free farmers from deep farmer-land attachment to realize permanent labor migration.”

2.In the introduction section, first paragraph, line number 4- Expand NBS and put NBS within brackets.

**Response to the comments:**

**Thank you for this suggestion—we have corrected the revised manuscript accordingly. The corresponding revisions are as follows:**
“However, according to the National Bureau of Statistics (NBS) in China, the urban-rural income gap (URIG) has maintained a high-level in recent years.”

3. The second last sentence of the second paragraph in the introduction section- The sentence starting with ‘Human capital theory partially………’ may be rewritten to convey the meaning correctly.

**Response to the comments:**

**Thank you for pointing out our problems. We have rearranged our introduction and deleted** **the paragraphs that were not closely related to the subject of the article, clearly indicated the background of our study, reasons for undertaking this study, related literature reviewed, and the significance of the study. In the revised manuscript, we have rewritten those appropriate sentences to convey the meaning correctly. Please see red area of the revised manuscript.**

4. Last sentence of the third paragraph in the introduction section- The sentence starting with ‘The fact indicates that………’ may be rewritten. It is not clear which fact……consider modifying the sentence to ‘This fact………….’

**Response to the comments:**

**Thank you for this suggestion—we have corrected the revised manuscript accordingly. The corresponding revisions are as follows:**
“This fact indicates that the hukou system has limited explanatory power for the current large-income gap between urban and rural residents in China, and it is essential to seek a new theoretical foundation to explain the factors in restricting labor mobility.”

5. Rewrite the first sentence of section 2.1

**Response to the comments:**

**Thank you for this suggestion—we have corrected the revised manuscript accordingly. The corresponding revisions are as follows:**
“Farmland finance is an important part of rural finance, and greatly affects urban and rural residents’ income distribution.”

6. Mention the citation of Greenwood et al correctly in section 2.1.2

**Response to the comments:**

**Thank you for pointing out this wrong citation. We carefully read the formatting sample and readjust all citations of the paper according to the guidelines for citations and referencing in the revised manuscript.**

7. Table 1- It is advisable to mention the source of data for individual parameters

**Response to the comments:**

**Thank you for reminding! We have supplemented the data source of the individual parameters in section 3.3 of the revised manuscript. The corresponding revisions are as follows:**

“The urban-rural income gap index is obtained from China Statistical Yearbook. The sub-indicators of the financial farmland innovation index are manually collected from the China Finance Statistical Yearbook and China Rural Finance Statistical Yearbook. Other provincial control variables are retrieved from the statistical yearbook of provinces and cities and the China Urban-Rural Construction Statistical Yearbook. The GDP data are retrieved from the website <http://www.stats.gov.cn/>.”

8. Section 3.2 Estimation formulas need to be incorporated as equations instead of explaining formulas in the sentence from

**Response to the comments:**

**Thank you for this suggestion—we have rewritten the estimation formulas as equations. (See red area in section 3.1.3)**

**The corresponding revisions are as follows:**

“The formula for measuring the amount of labor migration is as follows:”

$$Trans_{t}=Population_{t+1}-Population_{t}-Deaths_{t}+Newborn_{t}$$

$$Deaths_{t}=Population_{t}\times Deathsrate_{t}$$

$$Newborn_{t}=Population_{t}\times Birthrate_{t}$$

9. Table 3 - It is advisable to mention the source of data for individual parameters
**Response to the comments:**

**Thank you for reminding! We have supplemented the data source of the individual parameters in section 3.3 of the revised manuscript. The corresponding revisions are as follows:**

“We select 30 provinces (cities) in China (Tibet Province is not included) as the research sample. All raw data are collected at an annual frequency from 2006 to 2018. The urban-rural income gap index is obtained from China Statistical Yearbook. The sub-indicators of the financial farmland innovation index are manually collected from the China Finance Statistical Yearbook and China Rural Finance Statistical Yearbook. Other provincial control variables are retrieved from the statistical yearbook of provinces and cities and the China Urban-Rural Construction Statistical Yearbook. The GDP data are retrieved from the website http://www.stats.gov.cn/. We have described the data processing in detail in Section 3.1.”

10. Section 5.2.1. The term ‘negative correlation’ is not appropriate, consider revising it.

**Response to the comments:**

**Thank you for your suggestion. We have paid high attention to those expressions that might be inappropriate, and have followed your suggestions to make careful revisions to language. The corresponding revisions are as follows:**
“The baseline regression model has confirmed that farmland financial innovation has a negative effect on the urban-rural income gap.”

11. Section 5.2.1 first paragraph. Cite Baron et al (1986) as per the journal requirement.
**Response to the comments:**

**Thank you for reminding. We carefully read the formatting sample and readjust all citations of the paper according to the guidelines for citations and referencing.**

12. Section 5.4. Correct the spelling (of data) in the first sentence

**Response to the comments:**

**Thank you for your suggestion. We are sorry for this wrong spelling. Referring to the suggestions of other reviewers, we delete the sections that are not closely related to the theme of the article in the revised manuscript and try to give more important findings. Considering that the relationship between the impact of farmland financial innovation on urban-rural income gap and macroeconomic cycle (original section 5.4) has little relevance to the theme of this study, we cut out this part in the revised version. We also checked the spelling of our article carefully and we believe the spelling errors have been avoided in the** **revised manuscript.**

13. Terms like ‘As a matter of fact’ is not appropriate for research papers. Kindly consider revising.

**Response to the comments:**

**Thank you for your suggestion. We have revised those expressions that might be inappropriate, such as the terms you mentioned. The current manuscript has been copy-edited by American Journal Expert (AJE). We believe** **these mistakes has been dealt with in this version.**

14. Conclusion section- Rewrite initial sentences to remove similarity with the abstract
**Response to the comments:**

**Thank the reviewer very much for your suggestion and comment. We rewrite the sentences in conclusion section to remove similarity with the abstract. For your convenience, we have listed the corresponding revisions as follows:**

“Farmland financial innovation in China is a process of issuing bonds with the future income of land contract-management rights as a guarantee, thus transforming land into financial products that can be circulated in the financial market without losing land contract rights. Farmland financialization turns immovable land into an asset that can be "carried", liberating farmers from land attachment, and promoting farmers migrating to cities. Hence, we hold that farmland financial innovation is key to narrowing the income gap of residents between urban and rural areas.

This study examines the interaction relationships between farmland financial innovation and the urban-rural income gap using a panel data model over the period of 2006-2017. The main findings are as follows. The results show that the coefficient for farmland financial innovation is significantly negative at the 1% level, signifying that farmland financial innovation can significantly narrow the income gap between urban and rural areas, which also means that the poverty reduction effect of rural finance development is greater than the threshold effect. The findings of the mediating effect indicate that promoting the permanent migration of the labor force and upgrading the industrial structure are the potential transmission channels through which the farmland financial innovation narrows the urban-rural income gap.”

15. Try to give suggestions for future studies in this line

**Response to the comments:**

**Thanks for your advice. We have added necessary discussions regarding the future studies in the field of agricultural land finance research in the last paragraph of the conclusion section.**

“Future studies may try to develop a dual economy transformation model to understand the mechanism and impact of financial farmland innovation on labor migration and urban-rural inequality. Attention should also be paid to how different financial instruments contribute to reducing income inequality and which financial instrument works best.”

16. (Zhou and Cheung, 2017) is missing in the reference list.

**Response to the comments:**

**Thank you for pointing out this omission. We now add Zhou and Cheung (2017) in the reference list and we also check other references to ensure the accuracy and correctness.**

**Finally, we would like to express our gratitude to the editor and the three reviewers for the extremely helpful comments and for your guidance in the revision. We hope that our efforts have succeeded in allaying your concerns. We look forward to learning about your decision. And we express our thanks again to the referee for his time and efforts in reviewing our paper. Any remaining errors are our own.**
